# Synthesis, biological evaluation, and molecular docking of novel ferulic acid derivatives containing a 1,3,4-oxadiazole thioether and trifluoromethyl pyrimidine skeleton†

**DOI:** 10.1039/d4ra01765j

**Published:** 2024-05-20

**Authors:** Jiansong An, Nianjuan Pan, Chunyi Liu, Haijiang Chen, Qiang Fei, Xiuhai Gan, Wenneng Wu

**Affiliations:** a School of Food Science and Engineering, Guiyang University Guiyang 550005 China wuwenneng123@126.com; b National Key Laboratory of Green Pesticide, Key Laboratory of Green Pesticide and Agricultural Bioengineering, Ministry of Education, Guizhou University Guiyang 550025 China

## Abstract

In this study, 24 novel ferulic acid derivatives containing 1,3,4-oxadiazole thioether and trifluoromethyl pyrimidine were designed and synthesized. Bioactivity assay showed that some of the target compounds exhibited moderate to good antifungal activity against *Botryosphaeria dothidea* BD), *Phomopsis* sp. (PS), *Botrytis cinerea* (BC), *Fusarium* spp. (FS), *Fusarium graminearum* (FG), and *Colletotrichum* sp. (CS). Especially, compound 6f demonstrated superior antifungal activity against *Phomopsis* sp., with an EC_50_ value of 12.64 μg mL^−1^, outperforming pyrimethanil (35.16 μg mL^−1^) and hymexazol (27.01 μg mL^−1^). Meanwhile, compound 6p showed strong antibacterial activity against *X. axonopodis* pv. citri (XAC) *in vitro*, with an inhibition ratio of 85.76%, which was higher than thiodiazole copper's 76.59% at 100 μg mL^−1^. Furthermore, molecular docking simulations elucidated that compound 6f engaged in hydrogen bonding with the succinate dehydrogenase (SDH) enzyme at SER-17, SER-39, ARG-14 and ARG-43 sites, clarifying its mode of action. This study highlights the potential of these novel ferulic acid derivatives as promising agents for controlling fungal and bacterial threats to plant health. To the best of our knowledge, this study represents the first report on the antifungal and antibacterial properties of ferulic acid derivatives containing 1,3,4-oxadiazole thioether and trifluoromethyl pyrimidine skeleton.

## Abstract 2

Compound 6f displayed better antifungal activity against *Phomopsis* sp. Our study demonstrated that compound 6f formed hydrogen bonds with SER-17, SER-39, ARG-14 and ARG-43 of SDH.

## Graphical abstract

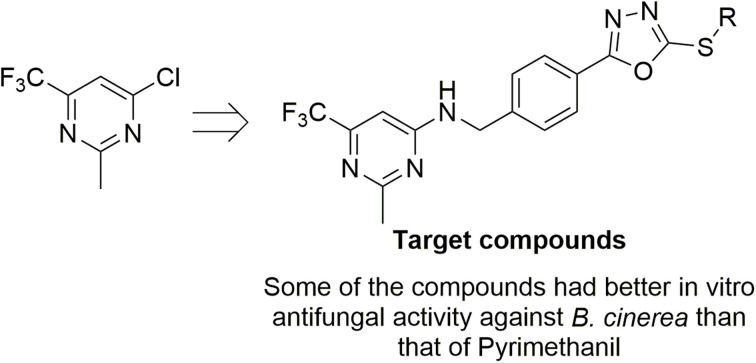

## 1. Introduction

Microbe-related diseases have posed a persistent threat to the healthy growth of crops, as well as to the overall quality and safety of agricultural produce.^1^ While pesticides remain a crucial component in the arsenal against these diseases in modern farming practices, their indiscriminate and prolonged use has given rise to two significant challenges.^2–4^ Firstly, it has led to the rapid development of resistance among pathogens, rendering many pesticides less effective. Secondly, the environmental pollution caused by these chemicals has become a cause for serious concern. In light of these issues, the quest for novel, highly effective, and environmentally benign antimicrobes has become an urgent priority.

Natural products are not only diverse and bioactive, but also have a unique mechanism of action, easy degradation and good environmental compatibility, which has made them important precursors for the development of chemical pesticides.^5–8^ Among them, ferulic acid is a natural plant metabolite that is usually found in *Ferula assa-foetida* L., *Ligusticum chuanxiong* Hort., *Catalpa ovata* G. Don^9^ and so on. Ferulic acid is a phenylpropanoid natural product containing a phenolic hydroxyl group and has a great variety of pharmacological activities, such as herbicidal,^10,11^ fungicidal,^12,13^ antiviral,^14,15^ and bactericidal^16–18^ activities. Therefore, ferulic acid derivatives are a class of lead compounds designed and synthesized for new pesticides.

**Fig. 1:**
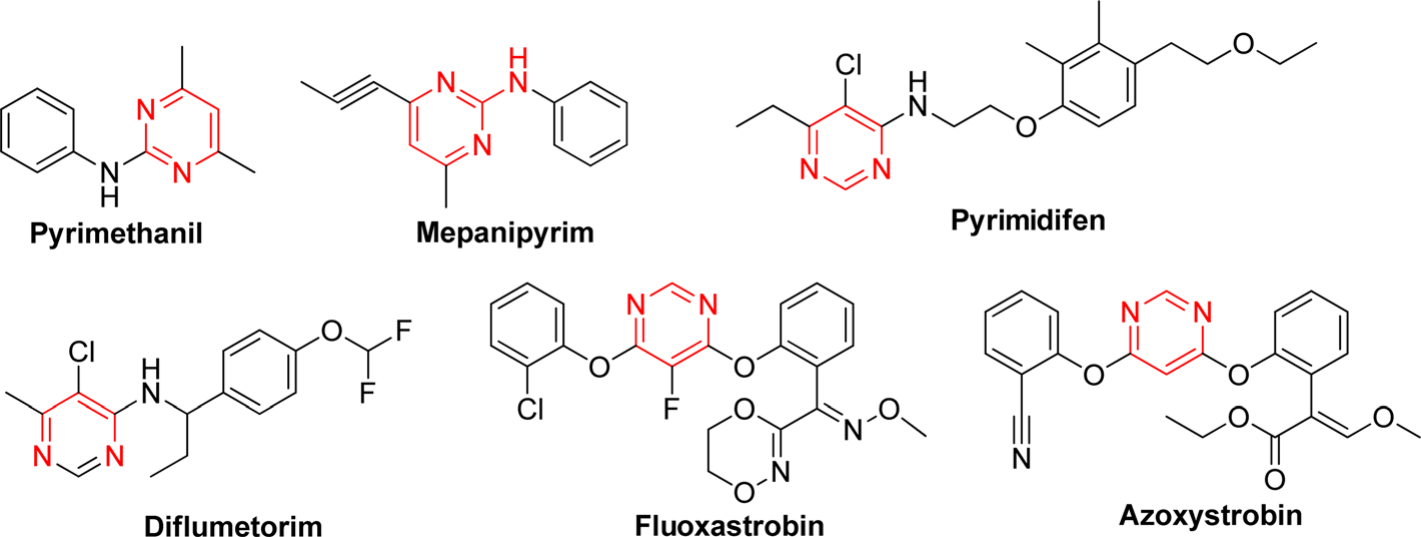
Some commercial fungicides with pyrimidine-based structures are commonly used.

Heterocyclic compounds have developed rapidly due to their high efficiency, low toxicity and broad-spectrum biological activity. Those compounds were not only widely used in the medical field, able to treat various diseases, but also in other fields such as agriculture, chemical industry and other applications. Among them, 1,3,4-oxadiazole stands out for its remarkable activity, selectivity, and low toxicity. In particularly, their thioether derivatives have found widespread applications in both medicine and pest control. 1,3,4-Oxadiazole thioether exhibits a broad spectrum of bioactivities, including insecticidal,^19,20^ fungicidal,^21,22^ herbicidal,^23^ antibacterial,^24,25^ and antiviral^26^ properties, as documented in various studies. In addition, pyrimidine was a crucial class of substances that play a significant role in various life activities, being prevalent in both human and other biological organisms. Due to their versatility, pyrimidine compounds found extensive applications in medicine and pesticides, with certain compounds even evolving into commercially viable fungicides (Fig. 1). Notably in recent years, a significant amount of studies had shown that pyrimidine derivatives have antifungal,^22,27^ antibacterial,^28^ insecticidal,^29^ herbicidal,^30^ antiviral^31^ properties, and so on. Especially trifluoromethyl pyrimidine derivatives exhibited excellent antifungal activity in our previous research.^32–38^

**Fig. 2:**
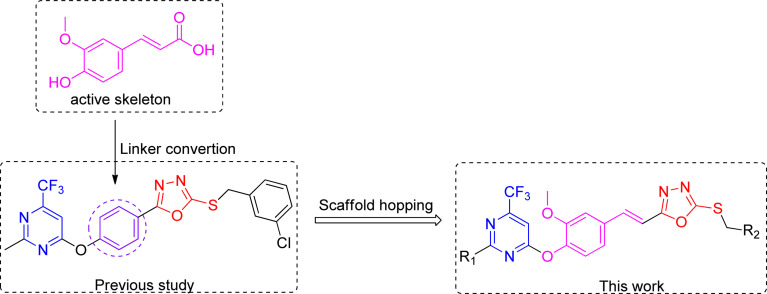
Molecular design for ferulic acid derivatives containing 1,3,4-oxadiazole thioether and trifluoromethyl pyrimidine rings.

Therefore, it is anticipated that the amalgamation of natural product ferulic acid with 1,3,4-oxadiazole thioether and pyrimidine moieties could lead to developing potent and eco-friendly antifungal and antibacterial agents. Currently, a range of ferulic acid derivatives incorporating 1,3,4-oxadiazole thioether and trifluoromethyl pyrimidine frameworks (Fig. 2) have been devised and synthesized. Subsequently, their antifungal and antibacterial efficacies were being assessed.

## 2. Materials and methods

### 2.1. Instruments and chemicals

Chemical reagents were sourced from Aladdin Reagent and Energy Chemical, both located in Shanghai, China. The melting points were measured with the aid of an XT-4 binocular microscope (Shanghai Electrophysics Optical Instrument Co., LTD.) NMR spectra, including ^1^H and ^13^C, were acquired on a Bruker 600 spectrometer. The title compounds were analyzed using a Thermo Scientific Q-Exactive instrument to acquire high-resolution mass spectrometry (HRMS) data.

### 2.2. Chemical synthesis

#### 2.2.1 Preparation procedure of (*E*)-3-(4-acetoxy-3-methoxyphenyl)acrylic acid (intermediates 2)

**Scheme 1:**
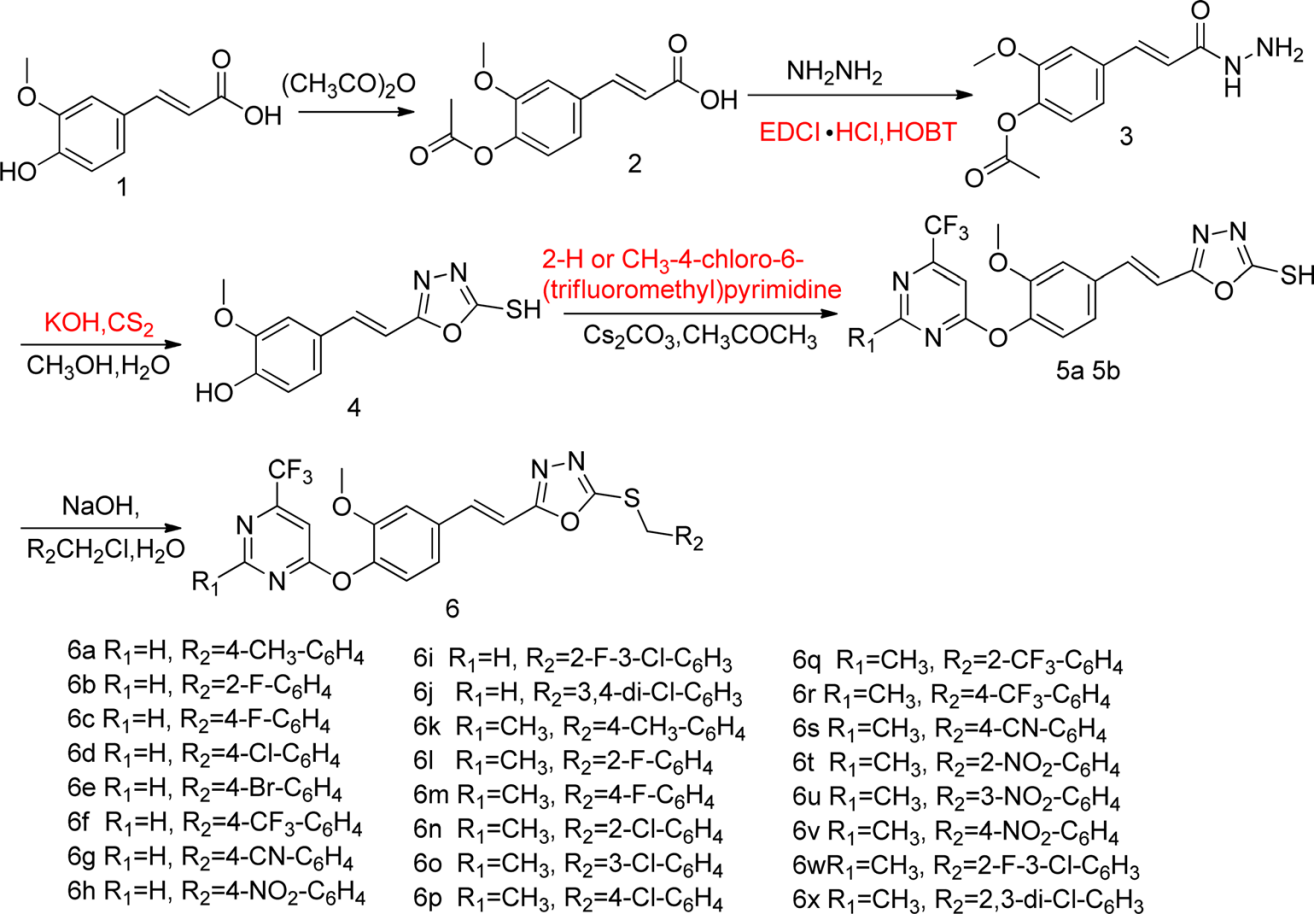
Synthesis route of target compounds 6a–6x.

As shown in Scheme 1, in a 500 mL three-neck flask, 50 mmol of intermediate 1 was mixed with 20 mmol of acetic anhydride and refluxed for a duration of 4 hours. After the reaction came to a halt, the mixture was gradually cooled until it reached room temperature. Subsequently, the solid residue underwent filtration, followed by drying. It was then recrystallized using anhydrous ethanol, ultimately yielding intermediate 2.

#### 2.2.2 Preparation procedure of (*E*)-4-(3-hydrazinyl-3-oxoprop-1-en-1-yl)-2-methoxyphenyl acetate (intermediates 3)

In a 250 mL three-necked flask, intermediate 3 (30 mmol), EDCI·HCl (30 mmol), HOBT (30 mmol), and 120 mL acetonitrile were prepared and stirred for 30 min at room temperature.^39^ Subsequently, 98% hydrazine hydrate (36 mmol) was gradually introduced into the reaction mixture using a constant pressure drop funnel, and the reaction was allowed to proceed at ambient temperature for 10 hours. Upon completion of the reaction, the solid residue was filtered, dried under vacuum conditions, and recrystallized from anhydrous ethanol to yield the desired intermediate 3.

#### 2.2.3 Preparation procedure of (*E*)-4-(2-(5-mercapto-1,3,4-oxadiazol-2-yl)vinyl)-2-methoxyphenol (intermediates 4)

In a 250 mL round-bottom flask, intermediate 3 (20 mmol) was combined with KOH (20 mmol) and anhydrous ethanol (60 mL). The mixture was stirred appropriately before carbon disulfide (25 mmol) was gradually added. The reaction was refluxed for 6 hours.^39^ Upon completion, the solid was recovered using spin-drying, diluted with 50 mL of distilled water, and filtered to remove insoluble matter. Subsequently, the filtrate was acidified with 5% HCl to a pH range of 2–3, resulting in the formation of a white solid. This solid was filtered, dried, and recrystallized from ethanol to yield the crucial intermediate 4.

##### (*E*)-4-(2-(5-Mercapto-1,3,4-oxadiazol-2-yl)vinyl)-2-methoxyphenol (4)

White solid; yield 80.5%; m.p. 219.2–220.4 °C; ^1^H NMR (600 MHz, DMSO-*d*_6_) *δ* 9.05 (s, Ph–OH), 7.48 (d, 1H, *J* = 16.20 Hz, –CH=CH–), 7.31 (s, 1H, Ph–H), 7.08 (d, 1H, *J* = 7.60 Hz, Ph–H), 7.08 (d, 1H, *J* = 16.20 Hz, –CH=CH–), 6.84 (s, 1H, *J* = 7.45 Hz, Ph–H), 3.79 (s, 3H, CH_3_O–).

#### 2.2.4 Preparation procedure of (*E*)-5-(3-methoxy-4-((2-methyl-6-(trifluoromethyl)pyrimidin-4-yl)oxy)styryl)-4*H*-1,2,4-triazole-3-thiol (intermediates 5a or 5b)

In a 100 mL three-necked flask, (*E*)-4-(2-(5-mercapto-1,3,4-oxadiazol-2-yl)vinyl)-2-methoxyphenol (10 mmol), cesium carbonate (12 mmol), and acetone (30 mL) were combined under ice-bath conditions. This mixture was stirred for 30 minutes while maintaining the ice bath conditions.^32^ Following this, 4-chloro-6-(trifluoromethyl)pyrimidine or 2-CH_3_-4-chloro-6-(trifluoromethyl)pyrimidine (8 mmol) dissolved in 5 mL of acetone was gradually introduced into the flask while maintaining the ice-bath conditions. The reaction mixture was then stirred continuously for 8 hours in the ice bath. Upon completion of the reaction, excess acetone was evaporated using a rotary evaporator, and the residue was extracted with ethyl acetate. The organic layer underwent rotary drying and subsequent purification *via* silica gel column chromatography, obtaining intermediates 5a and 5b.

##### (*E*)-5-(3-Methoxy-4-((2-methyl-6-(trifluoromethyl)pyrimidin-4-yl)oxy)styryl)-1,3,4-oxadiazole-2-thiol (5a)

White solid; yield 48.1%; m.p. 172.4–174.1 °C; ^1^H NMR (600 MHz, DMSO-*d*_6_) *δ*: 8.93 (s, 1H, pyrimidine-H), 7.82 (s, 1H, pyrimidine-H), 7.52 (d, 1H, *J* = 16.64 Hz, –CH=CH–), 7.30 (d, 1H, *J* = 1.56 Hz, Ph–H), 7.08 (dd, 1H, *J*_1_ = 8.16 Hz, *J*_2_ = 1.56 Hz, Ph–H), 7.06 (d, 1H, *J* = 16.64 Hz, –CH=CH–), 6.84 (s, 1H, *J* = 8.1 Hz, Ph–H), 3.80 (s, 3H, CH_3_O–).

##### (*E*)-5-(3-Methoxy-4-((2-methyl-6-(trifluoromethyl)pyrimidin-4-yl)oxy)styryl)-1,3,4-oxadiazole-2-thiol (5b)

White solid; yield 50.8%; m.p. 160.7–163.5 °C; ^1^H NMR (600 MHz, DMSO-*d*_6_) *δ*: 7.62 (s, 1H, pyrimidine-H), 7.54 (d, 1H, *J* = 16.32 Hz, –CH=CH–), 7.30 (d, 1H, *J* = 1.56 Hz, Ph–H), 7.08 (dd, 1H, *J*_1_ = 8.16 Hz, *J*_2_ = 1.56 Hz, Ph–H), 7.04 (d, 1H, *J* = 16.38 Hz, –CH=CH–), 6.84 (s, 1H, *J* = 8.1 Hz, Ph–H), 3.86 (s, 3H, CH_3_O–), 2.50 (s, 3H, CH_3_–).

#### 2.2.5 Preparation procedure of the target compounds 6a–6x

The target compounds 6a–6x were synthesized according to a procedure documented in the literature.^38^ In a 50 mL round-bottom flask, intermediate 5 (10 mmol) was introduced, followed by the addition of NaOH (12 mmol) dissolved in 10 mL of water. After thorough mixing and complete dissolution, RCH_2_X (10 mmol) was incorporated into the flask. The reaction mixture was allowed to proceed at ambient temperature for a duration of 5 to 6 hours. Upon completion, a white solid precipitate was observed, which was subsequently filtered and dried. The purification step involved column chromatography, obtaining the desired target compounds 6a–6x.

##### (*E*)-2-(3-Methoxy-4-((6-(trifluoromethyl)pyrimidin-4-yl)oxy)styryl)-5-((4-methylbenzyl)thio)-1,3,4-oxadiazole (6a)

White solid; yield 60.8%; m.p. 120.4–122.5 °C; ^1^H NMR (DMSO-*d*_6_, 600 MHz) *δ*: 8.95 (s, 1H, pyrimidine-H), 7.84 (s, 1H, pyrimidine-H), 7.67 (d, 1H, *J* = 1.2 Hz, Ph–H), 7.59 (d, 1H, *J* = 16.8 Hz, –CH=CH–), 7.44 (d, 1H, *J* = 16.8 Hz, –CH=CH–), 7.41 (d, 1H, *J* = 1.2 Hz, Ph–H), 7.38 (d, 2H, *J* = 7.8 Hz, Ph–H), 7.33 (d, 1H, *J* = 8.4 Hz, Ph–H), 7.17 (d, 1H, *J* = 7.8 Hz, Ph–H), 4.54 (s, 2H, –CH_2_–), 3.79 (s, 3H, CH_3_O–), 2.28 (s, 3H, CH_3_–); ^13^C NMR (DMSO-*d*_6_, 150 MHz) *δ*: 170.25, 165.66, 163.21, 159.82, 156.15 (q, *J* = 35.55 Hz), 151.62, 141.85, 138.50, 137.62, 134.62, 133.80, 129.64, 129.46, 123.52, 122.01, 121.80 (q, *J* = 273.3 Hz), 112.25, 110.61, 106.10, 56.55, 36.18, 21.18. HRMS (ESI) calcd for C_24_H_19_F_3_N_4_O_3_SNa [M + Na]^+^: 523.10052, found: 523.10222.

##### (*E*)-2-((2-Fluorobenzyl)thio)-5-(3-methoxy-4-((6-(trifluoromethyl)pyrimidin-4-yl)oxy)styryl)-1,3,4-oxadiazole (6b)

White solid; yield 50.4%; m.p. 101.3–103.7 °C; ^1^H NMR (DMSO-*d*_6_, 600 MHz) *δ*: 8.95 (s, 1H, pyrimidine-H), 7.83 (s, 1H, pyrimidine-H), 7.67 (d, 1H, *J* = 1.2 Hz, Ph–H), 7.59 (d, 1H, *J* = 16.8 Hz, –CH=CH–), 7.57 (s, 1H, Ph–H), 7.45 (d, 1H, *J* = 16.2 Hz, –CH=CH–), 7.41–7.37 (m, 2H, Ph–H), 7.38 (d, 2H, *J* = 7.8 Hz, Ph–H), 7.33 (d, 1H, *J* = 8.4 Hz, Ph–H), 7.26 (t, 1H, *J* = 9.0 Hz, Ph–H), 7.22 (t, 1H, *J* = 7.8 Hz, Ph–H), 4.60 (s, 2H, –CH_2_–), 3.80 (s, 3H, CH_3_O–); ^13^C NMR (DMSO-*d*_6_, 150 MHz) *δ*: 170.24, 165.90, 162.66, 161.71 (d, *J* = 244.95 Hz), 159.81, 156.16 (q, *J* = 35.4 Hz), 151.63, 141.89, 138.64, 134.57, 131.96 (d, *J* = 2.85 Hz), 130.79 (d, *J* = 7.8 Hz), 125.10 (d, *J* = 3.0 Hz), 123.98 (d, *J* = 14.55 Hz), 123.51, 122.06, 121.80 (q, *J* = 273.0 Hz), 116.08 (d, *J* = 20.85 Hz), 112.21 (d, *J* = 243.45 Hz), 106.09, 56.54, 30.27. HRMS (ESI) calcd for C_23_H_16_F_4_N_4_O_3_SNa [M + Na]^+^: 527.07611, found: 527.07715.

##### (*E*)-2-((4-Fluorobenzyl)thio)-5-(3-methoxy-4-((6-(trifluoromethyl)pyrimidin-4-yl)oxy)styryl)-1,3,4-oxadiazole (6c)

White solid; yield 72.8%; m.p. 110.2–112.1 °C; ^1^H NMR (CDCl_3_-*d*, 600 MHz) *δ*: 8.87 (s, 1H, pyrimidine-H), 7.48–7.44 (m, 3H, Ph–H and –CH=CH–), 7.34 (s, 1H, Ph–H), 7.22 (d, 3H, *J* = 7.52 Hz, Ph–H and pyrimidine-H), 7.08 (t, 2H, *J* = 8.6 Hz, Ph–H), 7.03 (d, 1H, *J* = 16.4 Hz, –CH=CH–), 4.52 (s, 2H, –CH_2_–), 3.84 (s, 3H, CH_3_O–); ^13^C NMR (CDCl_3_-*d*, 150 MHz) *δ*: 170.02, 165.28, 163.50, 162.35 (d, *J* = 245.88 Hz), 157.30 (q, *J* = 36.0 Hz), 151.54, 141.90, 137.73, 134.27, 131.43 (d, *J* = 2.35 Hz), 130.94 (d, *J* = 8.25 Hz), 123.16, 121.25 (q, *J* = 273.17 Hz), 115.84 (d, *J* = 21.57 Hz), 113.03, 110.26, 105.17, 55.89, 36.05; HRMS (ESI) calcd for C_23_H_16_F_4_N_4_O_3_SNa [M + Na]^+^: 527.07581, found: 527.07715.

##### (*E*)-2-((4-Chlorobenzyl)thio)-5-(3-methoxy-4-((6-(trifluoromethyl)pyrimidin-4-yl)oxy)styryl)-1,3,4-oxadiazole (6d)

White solid; yield 58.8%; m.p. 106.8–108.3 °C; ^1^H NMR (CDCl_3_-*d*, 600 MHz) *δ*: 8.87 (s, 1H, pyrimidine-H), 7.48 (d, 1H, *J* = 16.4 Hz, –CH=CH–), 7.44 (d, 2H, *J* = 6.3 Hz, Ph–H), 7.36–7.34 (m, 3H, Ph–H), 7.29 (s, 1H, Ph–H), 7.22 (d, 3H, *J* = 8.4 Hz, Ph–H and pyrimidine-H), 7.02 (d, 1H, *J* = 16.4 Hz, –CH=CH–), 4.50 (s, 2H, –CH_2_–), 3.84 (s, 3H, CH_3_O–); ^13^C NMR (CDCl_3_-*d*, 150 MHz) *δ*: 170.02, 165.32, 163.36, 159.29, 155.54 (q, *J* = 36.62 Hz), 151.54, 141.91, 137.77, 134.26, 134.20, 134.13, 130.52, 129.01, 123.17, 122.04, 121.79 (q, *J* = 270.1 Hz), 120.87, 112.02, 110.24, 105.15, 56.89, 36.05. HRMS (ESI) calcd for C_23_H_16_ClF_3_N_4_O_3_SNa [M + Na]^+^: 543.04608, found: 543.04759. HRMS (ESI) calcd for C_23_H_15_ClF_4_N_4_O_3_SNa [M + Na]^+^: 561.03674, found: 561.03817.

##### (*E*)-2-((4-Bromobenzyl)thio)-5-(3-methoxy-4-((6-(trifluoromethyl)pyrimidin-4-yl)oxy)styryl)-1,3,4-oxadiazole (6e)

White solid; yield 42.6%; m.p. 107.1–109.5 °C; ^1^H NMR (CDCl_3_-*d*, 600 MHz) *δ*: 8.86 (s, 1H, pyrimidine-H), 7.60 (s, 1H, Ph–H), 7.48 (d, 1H, *J* = 16.4 Hz, –CH=CH–), 7.44 (d, 1H, *J* = 7.9 Hz, Ph–H), 7.36–7.34 (d, 2H, *J* = 8.6 Hz, Ph–H), 7.29 (s, 1H, Ph–H), 7.22 (d, 3H, *J* = 8.6 Hz, Ph–H and pyrimidine-H), 7.02 (d, 1H, *J* = 16.4 Hz, –CH=CH–), 4.47 (s, 2H, –CH_2_–), 3.84 (s, 3H, CH_3_O–); ^13^C NMR (CDCl_3_-*d*, 150 MHz) *δ*: 170.02, 165.45, 162.98, 159.28, 157.30 (q, *J* = 35.78 Hz), 151.55, 141.94, 137.92, 136.07, 134.21, 132.84, 132.39, 131.03, 130.74, 128.52, 123.18, 121.25 (q, *J* = 273.21 Hz), 120.88, 111.08, 110.16, 105.13, 56.90, 35.45. HRMS (ESI) calcd for C_23_H_16_F_3_BrN_4_O_3_S Na [M + Na]^+^: 586.99542, found: 586.99708.

##### (*E*)-2-(3-Methoxy-4-((6-(trifluoromethyl)pyrimidin-4-yl)oxy)styryl)-5-((4-(trifluoromethyl)benzyl)thio)-1,3,4-oxadiazole (6f)

White solid; yield 71.3%; m.p. 97.3–99.7 °C; ^1^H NMR (DMSO-*d*_6_, 600 MHz) *δ*: 8.95 (s, 1H, pyrimidine-H), 7.83 (s, 1H, pyrimidine-H), 7.75–7.72 (m, 4H, Ph–H), 7.66 (d, 1H, *J* = 1.2 Hz), 7.57 (d, 1H, *J* = 16.8 Hz, –CH=CH–), 7.44 (d, 1H, *J* = 16.8 Hz, –CH=CH–), 7.41 (dd, 1H, *J*_1_ = 6.6 Hz, *J*_2_ = 1.8 Hz, Ph–H), 7.33 (d, 1H, *J* = 8.4 Hz, Ph–H), 4.67 (s, 2H, –CH_2_–), 3.79 (s, 3H, CH_3_O–); ^13^C NMR (DMSO-*d*_6_, 150 MHz) *δ*: 170.24, 165.81, 162.92, 159.81, 156.15 (q, *J* = 34.95 Hz), 151.62, 142.25, 141.87, 138.59, 134.57, 130.34, 128.81 (q, *J* = 31.5 Hz), 125.92 (q, *J* = 31.6 Hz), 125.53 (q, *J* = 270.3 Hz), 123.50, 121.80 (q, *J* = 272.1 Hz), 112.23, 110.56, 106.09, 56.53, 35.52. HRMS (ESI) calcd for C_24_H_16_F_6_N_4_O_3_SNa [M + Na]^+^: 577.07251, found: 577.07395.

##### (*E*)-4-(((5-(3-Methoxy-4-((6-(trifluoromethyl)pyrimidin-4-yl)oxy)styryl)-1,3,4-oxadiazol-2-yl)thio)methyl)benzonitrile (6g)

White solid; yield 63.2%; m.p. 135.5–137.6 °C; ^1^H NMR (DMSO-*d*_6_, 600 MHz) *δ*: 8.94 (s, 1H, pyrimidine-H), 7.85 (d, 2H, *J* = 7.8 Hz, Ph–H), 7.84 (s, 1H, pyrimidine-H), 7.71 (d, 2H, *J* = 7.8 Hz, Ph–H), 7.66 (d, 1H, *J* = 1.8 Hz, Ph–H), 7.56 (d, 1H, *J* = 16.2 Hz, –CH=CH–), 7.43 (d, 1H, *J* = 16.8 Hz, –CH=CH–), 7.39 (d, 1H, *J* = 1.8 Hz), 7.33 (d, 1H, *J* = 8.4 Hz, Ph–H), 4.65 (s, 2H, –CH_2_–), 3.79 (s, 3H, CH_3_O); ^13^C NMR (DMSO-*d*_6_, 150 MHz) *δ*: 170.24, 165.83, 162.83, 159.81, 156.27 (q, *J* = 34.65 Hz), 151.62, 143.25, 141.88, 138.64, 134.56, 132.94, 130.52, 123.51, 122.04, 121.80 (q, *J* = 272.7 Hz), 119.11, 112.24, 110.93, 110.54, 106.09, 56.55, 35.67. HRMS (ESI) calcd for C_24_H_16_F_3_N_5_O_3_S Na [M + Na]^+^: 534.08032, found: 534.08182.

##### (*E*)-2-(3-Methoxy-4-((6-(trifluoromethyl)pyrimidin-4-yl)oxy)styryl)-5-((4-nitrobenzyl)thio)-1,3,4-oxadiazole (6h)

White solid; yield 70.2%; m.p. 141.6–143.4 °C; ^1^H NMR (CDCl_3_-*d*, 600 MHz) *δ*: 8.85 (s, 1H, pyrimidine-H), 8.22 (d, 1H, *J* = 8.8 Hz, Ph–H), 7.70 (d, 2H, *J* = 8.7 Hz, Ph–H), 7.48 (d, 1H, *J* = 16.4 Hz, –CH=CH–), 7.33 (s, 1H, Ph–H), 7.21 (d, 3H, *J* = 7.0 Hz, Ph–H and pyrimidine-H), 7.0 (d, 1H, *J* = 16.4 Hz, –CH=CH–), 4.58 (s, 2H, –CH_2_–), 3.83 (s, 3H, CH_3_O–); ^13^C NMR (CDCl_3_-*d*, 150 MHz) *δ*: 170.00, 165.57, 162.72, 159.26, 156.26 (q, *J* = 36.0 Hz), 151.56, 147.66, 143.37, 141.98, 138.07, 134.14, 130.10, 123.94, 123.18, 121.25 (q, *J* = 273.08 Hz), 120.89, 111.08, 110.03, 105.17, 56.90, 35.62. HRMS (ESI) calcd for C_23_H_17_F_3_N_5_O_5_S Na [M + Na]^+^: 554.12427, found: 554.07165.

##### (*E*)-2-((3-Chloro-2-fluorobenzyl)thio)-5-(3-methoxy-4-((6-(trifluoromethyl)pyrimidin-4-yl)oxy)styryl)-1,3,4-oxadiazole (6i)

White solid; yield 50.4%; m.p. 135.5–137.6 °C; ^1^H NMR (CDCl_3_-*d*, 600 MHz) *δ*: 8.86 (s, 1H, pyrimidine-H), 7.52–7.48 (m, 1H, Ph–H), 7.49 (d, 1H, *J* = 16.4 Hz, –CH=CH–), 7.40–7.34 (m, 1H, Ph–H), 7.34 (s, 1H, Ph–H), 7.24–7.20 (m, 3H, Ph–H and pyrimidine-H), 7.02 (d, 1H, *J* = 16.4 Hz, –CH=CH–), 4.56 (s, 2H, –CH_2_–), 3.84 (s, 3H, CH_3_O–); ^13^C NMR (CDCl_3_-*d*, 150 MHz) *δ*: 170.02, 165.45, 163.14, 159.28, 157.33 (d, *J* = 249.11 Hz), 157.28 (q, *J* = 36.02 Hz), 151.54, 141.92, 138.88, 134.24, 130.70, 129.67 (d, *J* = 2.48 Hz), 125.09 (d, *J* = 14.26 Hz), 124.74 (d, *J* = 4.70 Hz), 123.16, 121.46 (d, *J* = 17.45 Hz), 121.25 (q, *J* = 273.11 Hz), 120.87, 111.07, 110.17, 105.17, 56.89, 29.93. HRMS (ESI) calcd for C_23_H_15_ClF_4_N_4_O_3_SNa [M + Na]^+^: 561.03674, found: 561.03817.

##### (*E*)-2-((3,4-Dichlorobenzyl)thio)-5-(3-methoxy-4-((6-(trifluoromethyl)pyrimidin-4-yl)oxy)styryl)-1,3,4-oxadiazole (6j)

White solid; yield 71.5%; m.p. 92.5–94.7 °C; ^1^H NMR (DMSO-*d*_6_, 600 MHz) *δ*: 8.95 (s, 1H, pyrimidine-H), 7.84 (s, 1H, Ph–H), 7.78 (d, 1H, *J* = 1.86 Hz, Ph–H), 7.71 (d, 1H, *J* = 16.38 Hz, –CH=CH–), 7.64–7.62 (m, 2H, Ph–H and pyrimidine-H), 7.50–7.47 (m, 2H, Ph–H and –CH=CH–), 7.37 (dd, 1H, *J*_1_ = 6.72 Hz, *J*_2_ = 1.56 Hz, Ph–H), 7.32 (d, 1H, *J* = 8.16 Hz, Ph–H), 4.63 (s, 2H, –CH_2_–), 3.79 (s, 3H, CH_3_O–); ^13^C NMR (DMSO-*d*_6_, 150 MHz) *δ*: 170.26, 168.41, 163.65, 159.84, 156.13 (q, *J* = 35.26 Hz), 151.58, 141.54, 138.62, 138.59, 135.06, 131.54, 131.46, 131.19, 130.73, 129.92, 123.49, 121.81 (d, *J* = 273.26 Hz), 121.62, 118.59, 112.00, 106.12, 56.50, 36.40. HRMS (ESI) calcd for C_23_H_15_Cl_2_F_3_N_4_O_3_SNa [M + Na]^+^: 577.00702, found: 577.00862.

##### (*E*)-2-(3-Methoxy-4-((2-methyl-6-(trifluoromethyl)pyrimidin-4-yl)oxy)styryl)-5-((4-methylbenzyl)thio)-1,3,4-oxadiazole (6k)

White solid; yield 46.4%; m.p. 134.0–135.2 °C; ^1^H NMR (DMSO-*d*_6_, 600 MHz) *δ*: 7.68 (d, 1H, *J* = 1.8 Hz, Ph–H), 7.68 (d, 1H, *J* = 16.2 Hz, –CH=CH–), 7.55 (s, 1H, pyrimidine-H), 7.44 (d, 1H, *J* = 16.2 Hz, –CH=CH–), 7.41 (dd, 1H, *J*_1_ = 6.0 Hz, *J*_2_ = 1.8 Hz, Ph–H), 7.38 (d, 1H, *J* = 8.4 Hz, Ph–H), 7.31 (d, 1H, *J* = 7.8 Hz, Ph–H), 7.18 (d, 1H, *J* = 7.8 Hz, Ph–H), 4.54 (s, 2H, –CH_2_–), 3.80 (s, 3H, CH_3_O–), 2.50 (s, 3H, CH_3_–), 2.28 (s, 3H, CH_3_–); ^13^C NMR (DMSO-*d*_6_, 150 MHz) *δ*: 170.20, 169.72, 165.67, 163.20, 156.27 (q, *J* = 34.95 Hz), 151.67, 141.91, 138.50, 137.62, 134.45, 133.81, 129.64, 129.46, 123.50, 122.08, 121.83 (q, *J* = 273.75 Hz), 112.40, 110.55, 102.84, 56.60, 36.18, 25.88, 21.18.

##### (*E*)-2-((2-Fluorobenzyl)thio)-5-(3-methoxy-4-((2-methyl-6-(trifluoromethyl)pyrimidin-4-yl)oxy)styryl)-1,3,4-oxadiazole (6l)

White solid; yield 65.2%; m.p. 115.6–117.1 °C; ^1^H NMR (DMSO-*d*_6_, 600 MHz) *δ*: 7.68 (d, 1H, *J* = 1.2 Hz, Ph–H), 7.59–7.54 (m, 3H, Ph–H and pyrimidine-H), 7.45 (d, 1H, *J* = 16.2 Hz, –CH=CH–), 7.41–7.37 (m, 2H, Ph–H), 7.32 (d, 1H, *J* = 8.4 Hz, Ph–H), 7.26 (t, 1H, *J* = 9.6 Hz, Ph–H), 7.22 (t, 1H, *J* = 7.2 Hz, Ph–H), 4.60 (s, 2H, –CH_2_–), 3.80 (s, 3H, CH_3_O–), 2.50 (s, 3H, CH_3_–); ^13^C NMR (DMSO-*d*_6_, 150 MHz) *δ*: 170.20, 169.72, 165.92, 162.65, 161.70 (d, *J* = 241.95 Hz), 156.27 (q, *J* = 34.65 Hz), 151.67, 141.94, 138.66, 134.40, 131.97 (d, *J* = 3.3 Hz), 130.80 (d, *J* = 8.25 Hz), 125.12 (d, *J* = 3.45 Hz), 123.99 (d, *J* = 13.95 Hz), 123.51, 122.13, 121.82 (q, *J* = 272.7 Hz), 116.09 (d, *J* = 15.6 Hz), 112.36, 110.53, 102.84, 56.60, 35.30, 25.87. HRMS (ESI) calcd for C_24_H_18_F_4_N_4_O_3_SNa [M + Na]^+^: 541.09280, found: 541.09161.

##### (*E*)-2-((4-Fluorobenzyl)thio)-5-(3-methoxy-4-((2-methyl-6-(trifluoromethyl)pyrimidin-4-yl)oxy)styryl)-1,3,4-oxadiazole (6m)

White solid; yield 55.8%; m.p.113.2–115.8 °C. ^1^H NMR (DMSO-*d*_6_, 600 MHz) *δ*: 7.67 (d, 1H, *J* = 1.8 Hz, Ph–H), 7.58 (d, 1H, *J* = 16.2 Hz, –CH=CH–), 7.55 (t, 3H, *J* = 3.6 Hz, Ph–H and pyrimidine-H), 7.44 (d, 1H, *J* = 16.2 Hz, –CH=CH–), 7.40 (dd, 1H, *J*_1_ = 6.6 Hz, *J*_2_ = 1.2 Hz, Ph–H), 7.31 (d, 1H, *J* = 8.4 Hz, Ph–H), 7.18 (t, 2H, *J* = 9.0 Hz, Ph–H), 4.58 (s, 2H, –CH_2_–), 3.80 (s, 3H, CH_3_O–), 2.50 (s, 3H, CH_3_–); ^13^C NMR (DMSO-*d*_6_, 150 MHz) *δ*: 170.20, 169.72, 165.73, 163.08, 162.93, 161.31, 156.27 (q, *J* = 34.80 Hz), 151.66, 141.92, 138.54, 134.43, 133.36, 131.68, 131.62, 123.49, 122.08, 121.82 (q, *J* = 273.15 Hz), 115.95, 115.81, 112.38, 110.52, 102.82, 56.59, 35.49, 25.87.

##### (*E*)-2-((2-Chlorobenzyl)thio)-5-(3-methoxy-4-((2-methyl-6-(trifluoromethyl)pyrimidin-4-yl)oxy)styryl)-1,3,4-oxadiazole (6n)

White solid; yield 76.8%; m.p. 207.6–209.3 °C; ^1^H NMR (DMSO-*d*_6_, 600 MHz) *δ*: 7.68 (s, 1H, Ph–H), 7.65 (d, 1H, *J* = 7.32 Hz, Ph–H), 7.59 (d, 1H, *J* = 16.5 Hz, –CH=CH–), 7.55–7.52 (m, 2H, Ph–H and pyrimidine-H), 7.46 (d, 1H, *J* = 16.38 Hz, –CH=CH–), 7.39–7.34 (m, 3H, Ph–H), 7.32 (d, 1H, *J* = 8.16 Hz, Ph–H), 4.66 (s, 2H, –CH_2_–), 3.80 (s, 3H, CH_3_O–), 2.51 (s, 3H, CH_3_–); ^13^C NMR (DMSO-*d*_6_, 150 MHz) *δ*: 170.19, 169.72, 165.94, 162.65, 156.25 (q, *J* = 34.77 Hz), 151.66, 138.68, 134.40, 134.27, 133.81, 130.09, 130.50, 130.13, 127.99, 123.51, 122.15, 121.83 (q, *J* = 273.09 Hz), 112.34, 110.52, 102.85, 56.59, 34.59, 25.88. HRMS (ESI) calcd for C_24_H_18_ClF_3_N_4_O_3_SNa [M + Na]^+^: 557.06324, found: 557.06189.

##### (*E*)-2-((3-Chlorobenzyl)thio)-5-(3-methoxy-4-((2-methyl-6-(trifluoromethyl)pyrimidin-4-yl)oxy)styryl)-1,3,4-oxadiazole (6o)

White solid; yield 75.7%; m.p. 95.5–96.7 °C; ^1^H NMR (DMSO-*d*_6_, 600 MHz) *δ*: 7.67 (d, 1H, *J* = 1.32 Hz, Ph–H), 7.60 (s, 1H, Ph–H), 7.57 (d, 1H, *J* = 16.5 Hz, –CH=CH–), 7.54 (s, 1H, Ph–H), 7.48 (d, 1H, *J* = 7.32 Hz, Ph–H), 7.44 (s, 1H, pyrimidine-H), 7.41–7.35 (m, 4H, Ph–H and –CH=CH–), 7.32 (d, 1H, *J* = 8.16 Hz, Ph–H), 4.58 (s, 2H, –CH_2_–), 3.80 (s, 3H, CH_3_O–), 2.50 (s, 3H, CH_3_–); ^13^C NMR (DMSO-*d*_6_, 150 MHz) *δ*: 170.21, 169.74, 165.81, 162.99, 156.28 (q, *J* = 34.71 Hz), 151.68, 141.94, 139.85, 138.59, 134.43, 133.48, 130.92, 129.41, 128.26, 128.20, 123.66, 122.11, 121.84 (q, *J* = 272.80 Hz), 112.37, 110.53, 102.84, 56.60, 35.51, 25.88. HRMS (ESI) calcd for C_24_H_18_ClF_3_N_4_O_3_SNa [M + Na]^+^: 557.06324, found: 557.06165.

##### (*E*)-2-((4-Chlorobenzyl)thio)-5-(3-methoxy-4-((2-methyl-6-(trifluoromethyl)pyrimidin-4-yl)oxy)styryl)-1,3,4-oxadiazole (6p)

White solid; yield 57.8%; m.p. 127.4–129.2 °C; ^1^H NMR (CDCl_3_-*d*, 600 MHz) *δ*: 7.48 (d, 1H, *J* = 15.56 Hz, –CH=CH–), 7.44 (d, 1H, *J* = 7.4 Hz, Ph–H), 7.35 (d, 1H, *J* = 8.16 Hz, Ph–H), 7.20 (d, 3H, *J* = 5.8 Hz, Ph–H), 7.05 (s, 1H, pyrimidine-H), 7.01 (d, 1H, *J* = 16.4 Hz, –CH=CH–), 4.50 (s, 2H, –CH_2_–), 3.83 (s, 3H, CH_3_O–), 2.63 (s, 3H, CH_3_–); ^13^C NMR (CDCl_3_-*d*, 150 MHz) *δ*: 169.99, 169.90, 165.37, 163.32, 157.31 (q, *J* = 35.42 Hz), 151.69, 142.19, 137.88, 1134.22, 134.12, 133.87, 130.52, 129.01, 123.25, 121.35 (q, *J* = 273.08 Hz), 120.89, 111.11, 110.03, 101.54, 55.93, 36.06, 25.79. HRMS (ESI) calcd for C_24_H_18_ClF_3_N_4_O_3_SNa [M + Na]^+^: 557.06324, found: 557.06140.

##### (*E*)-2-(3-Methoxy-4-((2-methyl-6-(trifluoromethyl)pyrimidin-4-yl)oxy)styryl)-5-((2-(trifluoromethyl)benzyl)thio)-1,3,4-oxadiazole (6q)

White solid; yield 66.5%; m.p. 95.5–97.3 °C; ^1^H NMR (DMSO-*d*_6_, 600 MHz) *δ*: 7.81 (t, 2H, *J* = 7.8 Hz, Ph–H), 7.72 (t, 1H, *J* = 7.8 Hz, Ph–H), 7.67 (d, 1H, *J* = 1.2 Hz, Ph–H), 7.58–7.52 (m, 3H, –CH=CH–, Ph–H and pyrimidine-H), 7.45 (d, 1H, *J* = 16.2 Hz, –CH=CH–), 7.39 (dd, 1H, *J*_1_ = 6.6 Hz, *J*_2_ = 1.8 Hz, Ph–H), 7.31 (d, 1H, *J* = 8.4 Hz, Ph–H), 4.73 (s, 2H, –CH_2_–), 3.79 (s, 3H, CH_3_O–), 2.49 (s, 3H, CH_3_–); ^13^C NMR (DMSO-*d*_6_, 150 MHz) *δ*: 170.18, 169.72, 165.98, 162.52, 156.27 (q, *J* = 34.65 Hz), 151.67, 141.96, 138.70, 134.70, 134.36, 133.55, 132.54, 129.25, 127.95 (q, *J* = 29.55 Hz), 126.86, 125.62 (q, *J* = 272.4 Hz), 123.49, 122.12, 121.82 (q, *J* = 273.0 Hz), 112.35, 110.48, 102.78, 56.57, 33.53, 25.83. HRMS (ESI) calcd for C_25_H_18_F_6_N_4_O_3_SNa [M + Na]^+^: 591.08960, found: 591.08826.

##### (*E*)-2-(3-Methoxy-4-((2-methyl-6-(trifluoromethyl)pyrimidin-4-yl)oxy)styryl)-5-((4-(trifluoromethyl)benzyl)thio)-1,3,4-oxadiazole (6r)

White solid; yield 47.8%; m.p. 116.3–117.9 °C; ^1^H NMR (DMSO-*d*_6_, 600 MHz) *δ*: 7.75–7.72 (m, 4H, Ph–H), 7.67 (d, 1H, *J* = 1.8 Hz, Ph–H), 7.56 (d, 1H, *J* = 16.2 Hz, –CH=CH–), 7.53 (s, 1H, pyrimidine-H), 7.44 (d, 1H, *J* = 16.2 Hz, –CH=CH–), 7.39 (dd, 1H, *J*_1_ = 6.6 Hz, *J*_2_ = 1.8 Hz, Ph–H), 7.31 (d, 1H, *J* = 8.4 Hz, Ph–H), 4.67 (s, 2H, –CH_2_–), 3.79 (s, 3H, CH_3_O–), 2.50 (s, 3H, CH_3_–); ^13^C NMR (DMSO-*d*_6_, 150 MHz) *δ*: 170.19, 169.71, 165.82, 162.91, 156.28 (q, *J* = 34.80 Hz), 151.66, 142.25, 141.93, 138.59, 134.40, 130.34, 128.82 (q, *J* = 31.65 Hz), 125.88, 125.53 (q, *J* = 270.6 Hz), 123.48, 122.08, 121.82 (q, *J* = 272.7 Hz), 112.37, 110.49, 102.81, 56.57, 35.53, 25.87. HRMS (ESI) calcd for C_25_H_18_F_6_N_4_O_3_SNa [M + Na]^+^: 591.08960, found: 591.08832.

##### (*E*)-4-(((5-(3-Methoxy-4-((2-methyl-6-(trifluoromethyl)pyrimidin-4-yl)oxy)styryl)-1,3,4-oxadiazol-2-yl)thio)methyl)benzonitrile (6s)

White solid; yield 52.1%; m.p. 132.9–134.7 °C; ^1^H NMR (DMSO-*d*_6_, 600 MHz) *δ*: 7.85 (d, 2H, *J* = 7.8 Hz, Ph–H), 7.71 (d, 2H, *J* = 8.4 Hz, Ph–H), 7.66 (d, 1H, *J* = 1.2 Hz, Ph–H), 7.56 (d, 1H, *J* = 16.2 Hz, –CH=CH–), 7.54 (s, 1H, pyrimidine-H), 7.43 (d, 1H, *J* = 16.8 Hz, –CH=CH–), 7.40 (dd, 1H, *J*_1_ = 6.6 Hz, *J*_2_ = 1.8 Hz, Ph–H), 7.31 (d, 1H, *J* = 8.4 Hz, Ph–H), 7.26 (t, 1H, *J* = 8.4 Hz, Ph–H), 4.65 (s, 2H, –CH_2_–), 3.79 (s, 3H, CH_3_O–), 2.50 (s, 3H, CH_3_–); ^13^C NMR (DMSO-*d*_6_, 150 MHz) *δ*: 170.19, 169.71, 165.85, 162.82, 156.27 (q, *J* = 34.65 Hz), 151.66, 143.24, 141.93, 138.64, 134.39, 132.94, 130.52, 123.49, 122.10, 121.82 (q, *J* = 273.45 Hz), 119.10, 112.38, 110.93, 110.48, 102.83, 56.59, 35.67, 25.87. HRMS (ESI) calcd for C_25_H_18_F_3_N_5_O_3_SNa [M + Na]^+^: 548.09747, found: 548.09570.

##### (*E*)-2-(3-Methoxy-4-((2-methyl-6-(trifluoromethyl)pyrimidin-4-yl)oxy)styryl)-5-((2-nitrobenzyl)thio)-1,3,4-oxadiazole (6t)

White solid; yield 73.9%; m.p. 112.6–114.5 °C; ^1^H NMR (CDCl_3_-*d*, 600 MHz) *δ*: 8.19 (d, 1H, *J* = 8.16 Hz, Ph–H), 7.91 (d, 1H, *J* = 7.68 Hz, Ph–H), 7.66 (t, 1H, *J* = 7.52 Hz, Ph–H), 7.55 (t, 1H, *J* = 8.16 Hz, Ph–H), 7.50 (d, 1H, *J* = 16.4 Hz, –CH=CH–), 7.22–7.18 (m, 3H, Ph–H), 7.04 (s, 1H, pyrimidine-H), 7.00 (d, 1H, *J* = 16.4 Hz, –CH=CH–), 4.88 (s, 2H, –CH_2_–), 3.83 (s, 3H, CH_3_O–), 2.62 (s, 3H, CH_3_–); ^13^C NMR (CDCl_3_-*d*, 150 MHz) *δ*: 169.98, 169.90, 165.50, 163.90, 157.29 (q, *J* = 35.45 Hz), 151.67, 147.68, 142.18, 137.93, 133.98, 133.85, 133.14, 132.61, 129.40, 125.64, 123.24, 121.35 (q, *J* = 273.06 Hz), 120.86, 112.16, 109.92, 101.52, 55.93, 34.26, 25.79. HRMS (ESI) calcd for C_24_H_18_F_3_N_5_O_5_S Na [M + Na]^+^: 568.08730, found: 568.08624.

##### (*E*)-2-(3-Methoxy-4-((2-methyl-6-(trifluoromethyl)pyrimidin-4-yl)oxy)styryl)-5-((3-nitrobenzyl)thio)-1,3,4-oxadiazole (6u)

White solid; yield 68.2%; m.p. 135.8–137.1 °C; ^1^H NMR (CDCl_3_-*d*, 600 MHz) *δ*: 8.39 (t, 1H, *J* = 1.48 Hz, Ph–H), 8.21 (dd, 1H, *J*_1_ = 6.88 Hz, *J*_2_ = 1.4 Hz, Ph–H), 7.90 (d, 1H, *J* = 7.68 Hz, Ph–H), 7.59 (t, 1H, *J* = 7.92 Hz, Ph–H), 7.50 (d, 1H, *J* = 16.4 Hz, –CH=CH–), 7.28 (s, 1H, Ph–H), 7.23–7.18 (m, 2H, Ph–H), 7.05 (s, 1H, pyrimidine-H), 7.02 (d, 1H, *J* = 16.4 Hz, –CH=CH–), 4.61 (s, 2H, –CH_2_–), 3.84 (s, 3H, CH_3_O–), 2.63 (s, 3H, CH_3_–); ^13^C NMR (CDCl_3_-*d*, 150 MHz) *δ*: 169.99, 169.89, 165.60, 162.70, 157.31 (q, *J* = 35.48 Hz), 151.70, 148.49, 142.24, 138.15, 138.11, 135.28, 133.78, 129.79, 124.02, 123.27, 123.14, 121.35 (q, *J* = 272.82 Hz), 120.88, 112.20, 109.88, 101.55, 55.94, 35.66, 25.79. HRMS (ESI) calcd for C_24_H_18_F_3_N_5_O_5_S Na [M + Na]^+^: 568.08730, found: 568.08612.

##### (*E*)-2-(3-Methoxy-4-((2-methyl-6-(trifluoromethyl)pyrimidin-4-yl)oxy)styryl)-5-((4-nitrobenzyl)thio)-1,3,4-oxadiazole (6v)

White solid; yield 44.9%; m.p. 133.6–135.7 °C; ^1^H NMR (CDCl_3_-*d*, 600 MHz) *δ*: 8.24 (d, 1H, *J* = 8.6 Hz, Ph–H), 7.70 (d, 2H, *J* = 8.6 Hz, Ph–H), 7.48 (d, 1H, *J* = 16.4 Hz, –CH=CH–), 7.19–7.18 (m, 3H, Ph–H), 7.05 (s, 1H, pyrimidine-H), 7.01 (d, 1H, *J* = 16.4 Hz, –CH=CH–), 4.59 (s, 2H, –CH_2_–), 3.83 (s, 3H, CH_3_O–), 2.62 (s, 3H, CH_3_–); ^13^C NMR (CDCl_3_-*d*, 150 MHz, 150 MHz) *δ*: 169.98, 169.88, 165.62, 162.68, 157.30 (q, *J* = 35.48 Hz), 151.70, 147.69, 143.34, 142.27, 138.17, 133.75, 130.10, 123.97, 123.27, 121.35 (q, *J* = 273.12 Hz), 120.92, 112.12, 109.83, 101.56, 55.93, 35.65, 25.79. HRMS (ESI) calcd for C_24_H_18_F_3_N_5_O_5_S Na [M + Na]^+^: 568.08730, found: 568.08582.

##### (*E*)-2-((3-Chloro-2-fluorobenzyl)thio)-5-(3-methoxy-4-((2-methyl-6-(trifluoromethyl)pyrimidin-4-yl)oxy)styryl)-1,3,4-oxadiazole (6w)

White solid; yield 58.4%; m.p. 115.7–117.1 °C; ^1^H NMR (DMSO-*d*_6_, 600 MHz) *δ*: 7.67 (d, 1H, *J* = 1.8 Hz, Ph–H), 7.57–7.54 (m, 4H, –CH=CH–, Ph–H and pyrimidine-H), 7.45 (d, 1H, *J* = 16.8 Hz, –CH=CH–), 7.45 (dd, 1H, *J*_1_ = 6.6 Hz, *J*_2_ = 1.2 Hz, Ph–H), 7.32 (d, 1H, *J* = 7.8 Hz, Ph–H), 7.26 (t, 1H, *J* = 8.4 Hz, Ph–H), 4.63 (s, 2H, –CH_2_–), 3.80 (s, 3H, CH_3_O–), 2.50 (s, 3H, CH_3_–); ^13^C NMR (DMSO-*d*_6_, 150 MHz) *δ*: 170.19, 169.72, 165.99, 162.43, 156.40 (d, *J* = 247.65 Hz), 156.27 (q, *J* = 34.65 Hz), 151.26, 151.67, 141.96, 138.71, 134.37, 130.87, 130.76, 126.37 (d, *J* = 14.1 Hz), 125.96 (d, *J* = 4.2 Hz), 123.51, 122.14, 121.82 (q, *J* = 273.15 Hz), 120.31 (d, *J* = 17.25 Hz), 112.35, 110.51, 102.84, 56.60, 30.18, 25.87. HRMS (ESI) calcd for C_24_H_17_ClF_4_N_4_O_3_SNa [M + Na]^+^: 575.05382, found: 575.05261.

##### (*E*)-2-((2,3-Dichlorobenzyl)thio)-5-(3-methoxy-4-((2-methyl-6-(trifluoromethyl)pyrimidin-4-yl)oxy)styryl)-1,3,4-oxadiazole (6x)

White solid; yield 51.7%; m.p. 122.8–125.0 °C; ^1^H NMR (DMSO-*d*_6_, 600 MHz) *δ*: 7.68 (s, 1H, Ph–H), 7.64 (t, 2H, *J* = 6.6 Hz, Ph–H), 7.58 (d, 2H, *J* = 16.8 Hz, –CH=CH– and pyrimidine-H), 7.45 (d, 1H, *J* = 16.8 Hz, –CH=CH–), 7.38 (t, 2H, *J* = 7.8 Hz, Ph–H), 7.31 (d, 1H, *J* = 8.4 Hz, Ph–H), 4.71 (s, 2H, –CH_2_–), 3.80 (s, 3H, CH_3_O–), 2.50 (s, 3H, CH_3_–); ^13^C NMR (DMSO-*d*_6_, 150 MHz) *δ*: 170.20, 169.72, 166.01, 162.45, 156.27 (q, *J* = 34.5 Hz), 151.67, 141.96, 138.75, 137.09, 134.39, 132.63, 131.88, 130.81, 130.67, 128.83, 123.52, 122.16, 121.83 (q, *J* = 273.45 Hz), 112.38, 110.51, 102.85, 56.61, 35.39, 25.88. HRMS (ESI) calcd for C_24_H_17_Cl_2_F_3_N_4_O_3_SNa [M + Na]^+^: 591.02427, found: 591.12203.

### 2.3. *In vitro* antifungal activity test

The antifungal activities of compounds 6a–6x were evaluated *in vitro* against a range of fungal pathogens, including *Botryosphaeria dothidea* (BD), *Phomopsis* sp. (PS), *Botrytis cinerea* (BC), *Fusarium* spp. (FS), *Fusarium graminearum* (FG), and *Colletotrichum* sp. (CS), utilizing a previously reported methodology.^36–38^ The target compound (5 mg) was dissolved in 1 mL of DMSO and then added into PDA plates at a final working concentration of 50 μg mL^−1^. Mycelial discs, measuring 0.4 cm in diameter, were carefully placed at the center of each plate under sterile conditions. These plates were then incubated at a constant temperature of 28 °C for 3–4 days. For accurate comparison, DMSO, pyrimethanil and hymexazol at equal concentrations were used respectively as negative controls and positive controls. The inhibition rate (*I*) was determined using the following formula, where *C* represents the fungal diameter on the untreated PDA plate (control), while *T* denotes the fungal diameter on the treated PDA plate (test sample). This method provides a quantitative measure of the antifungal activity of the target compounds.Inhibition rate *I* (%) = (*C* − *T*)/(*C* − 0.4) × 100

### 2.4. *In vitro* antibacterial activity test^39^

The antibacterial efficacy of compounds 6a–6y was examined *in vitro* against three significant plant pathogens: *Xanthomonas oryzae* pv. oryzicola (XOO), *X. axonopodis* pv. citri (XAC), and *Pseudomonas syringae* pv. actinidiae (Psa). Following a standardized procedure, each compound (3.75 mg) was dissolved in DMSO (150 μL) and subsequently diluted with a 0.1% aqueous Tween solution to formulate test solutions. These solutions were then combined with nutrient broth (NB) to attain final concentrations of 100 and 50 μg mL^−1^. Bacterial suspensions in NB (40 μL) were introduced into the test tubes, which were then incubated at 30 °C with continuous shaking at 180 rpm for 48 hours. Bacterial proliferation was assessed spectrophotometrically using a Multiskan Sky1530 spectrophotometer, relying on OD_595_ values within the 0.6 to 0.8 range to identify the logarithmic growth phase. DMSO and thiodiazole copper served as the negative and positive controls, respectively. The percentage inhibition (*I*%) was calculated based on adjusted turbidity readings from both treated and untreated NB samples.Inhibition rate *I* (%) = (*C* − *T*)/*C* × 100

### 2.5. Molecular docking

The enzyme SDH plays a crucial role in the Krebs cycle, making it a potential target for developing antifungal agents known as SDHIs. To understand its mechanisms and target interactions, we studied the binding patterns between SDH and the highly effective compound 6f using Discovery Studio 2.5 software. This molecular docking analysis provided insights into how compound 6f interacts with SDH, following methodologies outlined in previous studies.^37,38^

## 3. Result and discussion

### 3.1. Chemistry

Utilizing ferulic acid as the initial material, the title compounds 6a–6x were crafted *via* acetylation, condensation, cyclization, etherification, and thioetherification reactions, affording yields ranging from 42.5% to 84.6%. The chemical structures of these compounds were unambiguously verified using ^1^H NMR, ^13^C NMR, and HRMS techniques. In the case of compound 6b, its ^1^H NMR spectrum exhibited two distinctive singlets at 7.84 and 7.67 ppm, confirming the presence of a hydrogen atom within the pyrimidine moiety. Additionally, the characteristic twin peaks observed at 7.59 and 7.45 ppm correspond to the two hydrogens present on the –CH=CH– group. Moreover, the molecular weight of compound 6b was precisely determined using HRMS, revealing the presence of [M + Na]^+^ ions with an *m*/*z* value of 527.07715. This information not only complements the structural elucidation but also provides further evidence for the successful synthesis and characterization of the target compounds. The spectra of ^1^H NMR and ^13^C NMR for compounds 6a–6x are shown in ESI.†

### 3.2. *In vitro* antifungal activity test

As shown in Table 1, the target compounds 6a–6y exhibited certain *in vitro* antifungal activities against BD (4.61–52.87%), PS (16.90–91.61%), BC (21.05–57.93%), FS (8.31–75.60%), FG (6.32–54.98%), and CS (20.87–58.64%). Among them, the inhibitory rates of compounds 6c, 6f, 6g and 6h against PS were 80.33%, 91.61%, 84.49%, and 89.10% respectively, higher than that of pyrimethanil (77.17%). Additionally, compounds 6c and 6d demonstrated moderate inhibition rates of 75.6% and 72.16% against FS respectively, which were superior to hymexazol (60.31%).

**Table 1:** Antifungal activity of the target compounds against the test fungi at concentration of 50 μg mL^−1^^a^

	Inhibition rate (%)					
	BD	PS	BC	FS	FG	CS
6a	5.31 ± 1.67	57.35 ± 2.36	23.22 ± 2.45	17.58 ± 1.12	24.59 ± 2.57	38.95 ± 1.08
6b	5.69 ± 1.17	70.85 ± 1.39	32.11 ± 2.37	59.71 ± 1.16	22.30 ± 1.26	34.04 ± 1.16
6c	12.73 ± 1.39	80.83 ± 2.44	38.15 ± 2.29	75.60 ± 0.82	31.58 ± 2.63	58.64 ± 1.60
6d	52.87 ± 1.95	57.68 ± 2.29	30.02 ± 1.24	72.16 ± 1.41	26.48 ± 1.61	50.30 ± 1.67
6e	12.39 ± 1.81	47.85 ± 1.43	27.11 ± 2.39	57.12 ± 1.22	16.51 ± 3.40	28.51 ± 2.29
6f	23.15 ± 1.56	91.61 ± 3.12	31.27 ± 1.93	67.24 ± 1.24	18.19 ± 1.83	28.51 ± 1.16
6g	7.64 ± 1.92	84.49 ± 3.20	35.02 ± 2.21	52.73 ± 1.36	17.45 ± 2.27	21.91 ± 2.85
6h	12.20 ± 1.12	89.10 ± 2.36	20.44 ± 1.31	48.77 ± 2.34	22.49 ± 1.73	22.98 ± 1.29
6i	5.73 ± 2.85	68.02 ± 1.80	57.93 ± 2.35	8.72 ± 1.17	12.85 ± 1.66	23.83 ± 1.25
6j	16.77 ± 1.50	25.46 ± 1.72	47.10 ± 3.30	16.01 ± 1.16	21.60 ± 1.43	48.30 ± 2.42
6k	7.93 ± 2.12	49.98 ± 1.22	21.05 ± 1.59	13.34 ± 1.15	49.56 ± 1.52	37.99 ± 1.38
6l	16.90 ± 2.13	52.26 ± 1.32	35.02 ± 1.93	17.47 ± 2.16	20.38 ± 1.69	23.83 ± 1.51
6m	7.59 ± 2.35	62.62 ± 2.28	20.86 ± 1.95	18.35 ± 1.18	54.98 ± 1.21	35.85 ± 2.29
6n	11.40 ± 1.27	16.90 ± 1.12	24.81 ± 1.72	26.01 ± 1.72	18.39 ± 1.15	26.17 ± 1.71
6o	17.99 ± 2.47	34.12 ± 1.09	27.10 ± 1.79	43.36 ± 0.86	30.77 ± 2.01	28.51 ± 1.08
6p	23.50 ± 3.75	43.50 ± 1.30	43.50 ± 2.07	53.50 ± 1.59	33.50 ± 3.21	45.50 ± 1.38
6q	12.22 ± 1.24	56.33 ± 1.05	25.44 ± 1.78	26.47 ± 2.21	6.32 ± 1.99	28.51 ± 2.23
6r	18.23 ± 2.42	68.23 ± 1.42	29.19 ± 1.40	37.07 ± 1.32	15.75 ± 1.13	30.43 ± 1.08
6s	15.24 ± 1.92	64.31 ± 3.20	25.44 ± 2.21	37.83 ± 1.36	10.73 ± 2.27	27.02 ± 2.85
6t	24.94 ± 2.15	48.29 ± 1.26	20.86 ± 1.27	8.31 ± 1.92	20.31 ± 1.15	20.87 ± 2.42
6u	32.39 ± 1.37	58.47 ± 1.28	36.27 ± 2.43	16.77 ± 1.53	23.45 ± 1.05	30.64 ± 1.28
6v	41.57 ± 1.29	68.64 ± 1.26	45.86 ± 2.16	30.32 ± 1.30	31.84 ± 1.82	42.77 ± 1.16
6w	12.04 ± 2.53	48.72 ± 2.12	42.11 ± 1.66	11.83 ± 1.11	14.56 ± 2.37	27.23 ± 1.95
6x	4.61 ± 0.95	27.72 ± 1.23	33.47 ± 3.37	30.95 ± 3.76	16.51 ± 0.95	35.32 ± 1.77
Hymexazol	34.55 ± 1.08	47.09 ± 1.69	71.26 ± 4.43	60.31 ± 1.40	59.97 ± 1.38	30.83 ± 1.13
Pyrimethanil	87.16 ± 1.54	86.56 ± 1.76	81.99 ± 2.34	39.38 ± 1.68	26.89 ± 0.94	65.12 ± 1.54

a Average of three replicates.

The EC_50_ values of some target compounds against *Phomopsis* sp. and *Fusarium* spp. were determined and the results are shown in Table 2. The results presented in Table 2 demonstrate that compounds 6c, 6f, 6g, and 6h exhibited superior antifungal activity against *Phomopsis* sp., with EC_50_ values of 20.57, 12.64, 26.19 and 21.55 μg mL^−1^ respectively, outperforming pyrimethanil (35.16 μg mL^−1^) and hymexazol (27.01 μg mL^−1^). Meanwhile, compounds 6c and 6d displayed excellent antifungal activity against *Fusarium* spp., with EC_50_ values of 21.35 and 27.46 μg mL^−1^, respectively, which were better than hymexazol (30.79 μg mL^−1^).

**Table 2:** The EC_50_ values of some of the title compounds against *Phomopsis* sp. and *Fusarium* spp.^a^

Compd	PS		FS	
	Regression eq.	EC_50_ (μg mL^−1^)	Regression eq.	EC_50_ (μg mL^−1^)
6c	*y* = 1.244*x* + 4.332	20.57 ± 2.43	*y* = 0.873*x* + 3.452	21.35 ± 1.51
6d	—	—	*y* = 1.057*x* + 3.685	27.46 ± 1.28
6f	*y* = 0.935*x* + 3.146	12.64 ± 1.15	—	—
6g	*y* = 1.3950*x* + 3.853	26.19 ± 2.04	—	—
6h	*y* = 0.972*x* + 3.192	21.55 ± 1.66	—	—
Hymexazol	*y* = 0.744*x* + 3.933	27.01 ± 1.34	*y* = 1.184*x* + 3.238	30.79 ± 1.72
Pyrimethanil	*y* = 1.654*x* + 4.248	35.16 ± 1.95	—	—

a Average of three replicates.

### 3.3. *In vitro* antibacterial activity test

The *in vitro* antibacterial activities of compounds 6a–6x were assessed against plant pathogens XOO, XAC, and PSA using turbidimeter tests. Table 3 summarizes the preliminary results, revealing that some test compounds showed moderate antibacterial activity in comparison to Thiodiazole copper. Notably, compounds 6c, 6h, and 6p exhibited exceptional inhibitory activity against XAC, achieving inhibitory rates higher than the commercial control drug at both 100 μg mL^−1^ and 50 μg mL^−1^. Specifically, compounds 6c, 6h, and 6p achieved inhibitory rates of 79.33%, 71.93%, and 85.76%, respectively, compared to 76.59% for thiodiazole copper at 100 μg mL^−1^. Compounds 6c, 6n, and 6p demonstrated significant inhibitory activity with rates of 63.26%, 50.46%, and 57.85%, respectively, surpassing the 48.01% rate of thiodiazole copper at 50 μg mL^−1^. The other compounds displayed varying degrees of antibacterial activity against the tested pathogens.

**Table 3:** Antibacterial activity of the target compounds against the test bacteria at concentrations of 100 and 50 μg mL^−1^^a^

Compounds	Inhibition rate (%)					
	XOO		XAC		PSA	
	100 μg mL^−1^	50 μg mL^−1^	100 μg mL^−1^	50 μg mL^−1^	100 μg mL^−1^	50 μg mL^−1^
6a	18.93 ± 2.63	9.18 ± 1.71	34.41 ± 1.56	17.71 ± 3.36	16.53 ± 2.11	7.38 ± 2.20
6b	40.21 ± 3.71	18.54 ± 2.59	13.13 ± 1.34	10.06 ± 0.78	11.24 ± 2.31	8.31 ± 1.12
6c	26.40 ± 3.93	16.81 ± 3.41	79.33 ± 2.54	63.26 ± 1.56	8.49 ± 4.95	5.50 ± 4.30
6d	14.88 ± 4.36	0	48.34 ± 2.87	43.56 ± 3.80	9.39 ± 1.49	0
6e	22.04 ± 2.65	4.84 ± 2.04	58.89 ± 2.73	40.81 ± 2.16	12.10 ± 2.13	0
6f	6.38 ± 1.25	1.47 ± 1.71	41.58 ± 3.88	20.16 ± 1.35	15.95 ± 3.49	7.91 ± 1.71
6g	17.89 ± 3.43	8.49 ± 2.70	40.46 ± 1.25	26.64 ± 4.15	19.88 ± 1.94	11.05 ± 5.93
6h	47.89 ± 0.53	46.16 ± 4.69	71.93 ± 3.26	33.32 ± 3.03	39.28 ± 0.66	8.70 ± 2.91
6i	38.40 ± 2.05	17.98 ± 1.21	45.84 ± 3.28	22.13 ± 1.35	21.05 ± 2.49	10.83 ± 1.91
6j	16.03 ± 2.72	1.60 ± 0.19	14.45 ± 4.05	10.59 ± 1.63	23.10 ± 3.44	0
6k	54.27 ± 3.52	29.31 ± 1.11	18.46 ± 1.28	10.54 ± 0.95	28.94 ± 1.59	19.32 ± 1.71
6l	56.43 ± 0.65	30.25 ± 0.81	40.58 ± 2.28	20.06 ± 1.35	45.73 ± 3.47	19.85 ± 0.97
6m	36.64 ± 2.57	13.95 ± 4.42	51.85 ± 2.81	42.63 ± 3.03	36.34 ± 3.24	0
6n	30.66 ± 3.01	11.95 ± 4.47	68.91 ± 0.27	50.46 ± 1.31	35.89 ± 3.79	0
6o	15.48 ± 1.35	7.14 ± 3.41	52.99 ± 1.28	35.53 ± 1.38	17.08 ± 5.49	8.81 ± 1.15
6p	37.46 ± 1.07	10.34 ± 3.41	85.76 ± 0.09	57.85 ± 2.41	28.59 ± 1.43	0
6q	11.38 ± 0.35	7.73 ± 0.61	58.15 ± 2.18	34.73 ± 2.31	54.17 ± 3.49	24.91 ± 1.08
6r	5.60 ± 1.75	0.85 ± 1.91	41.38 ± 1.28	22.13 ± 1.37	23.58 ± 1.09	10.16 ± 3.11
6s	35.86 ± 1.25	17.34 ± 0.81	55.35 ± 1.18	36.13 ± 0.35	39.84 ± 2.38	19.91 ± 1.01
6t	40.28 ± 3.10	19.18 ± 0.92	46.22 ± 1.71	42.77 ± 1.90	35.32 ± 3.91	5.05 ± 1.16
6u	19.00 ± 4.35	13.73 ± 6.91	39.01 ± 8.98	37.03 ± 2.35	19.18 ± 5.49	4.41 ± 1.71
6v	24.50 ± 1.04	19.72 ± 6.78	58.31 ± 1.72	43.93 ± 3.44	29.59 ± 1.32	10.61 ± 1.55
6w	31.16 ± 0.33	15.63 ± 1.39	56.58 ± 1.26	32.55 ± 0.93	44.21 ± 1.56	20.22 ± 1.74
6x	44.38 ± 1.23	31.09 ± 2.17	18.33 ± 1.06	11.54 ± 1.03	45.38 ± 2.06	22.56 ± 1.21
Thiadiazole copper	61.36 ± 3.17	50.36 ± 3.72	76.59 ± 3.10	48.01 ± 2.33	92.67 ± 5.22	45.29 ± 3.25

a Average of three replicates.

### 3.4. Docking analysis

**Fig. 3:**
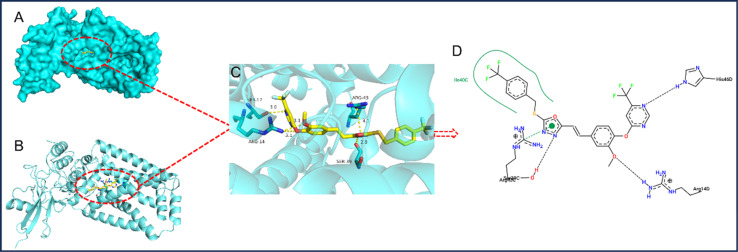
The molecular docking stimulation of compound 6f towards SDH.

The interaction between a potent compound and the SDH enzyme was investigated using docking techniques, with results shown in Fig. 3. The observed lowest binding energy was −9.8 kcal mol^−1^. In compound 6f, the oxygen atoms belonging to the pyrimidinyloxy ether and phenyloxy ether groups engage in a dual hydrogen bonding interaction with the ARG-14 residue, maintaining a consistent distance of 3.1 Å for both bonds. Otherwise, compound 6f utilizes nitrogen atoms on its pyrimidine and 1,3,4-oxadiazole rings to establish hydrogen bonds with SER-17 and SER-39 residues at distances of 3.0 Å and 2.8 Å, respectively. Additionally, the oxadiazole ring engages in a pi–cation interaction with ARG-43, involving electrostatic attraction. These findings enhance our understanding of the compound's interaction with SDH, revealing potential mechanisms of action and informing future therapeutic designs.

## 4. Conclusion

In this investigation, 24 innovative ferulic acid derivatives, containing 1,3,4-oxadiazole thioether and trifluoromethyl pyrimidine moieties, were devised and synthesized. Remarkably, compound 6f exhibited pronounced antifungal efficacy against *Phomopsis* sp., surpassing that of pyrimethanil and hymexazol in laboratory tests. This research provides a valuable framework for developing novel ferulic acid derivatives tailored for managing fungal and bacterial infections in plants. Furthermore, molecular docking simulations elucidated that compound 6f engages in hydrogen bonding with the SDH enzyme at SER-17, SER-39, ARG-14 and ARG-43 sites, clarifying its mode of action. Consequently, these ferulic acid derivatives arise as promising agents for tackling fungal and bacterial threats in plant health.

## Conflicts of interest

The authors declare no conflict of interest.

## Supplementary Material

RA-014-D4RA01765J-s001
