# Supplementary material for: Synthesis, biological evaluation, and molecular docking of novel ferulic acid derivatives containing a 1,3,4-oxadiazole thioether and trifluoromethyl pyrimidine skeleton
Source: RSC Adv. 2024 May 20;14(23):16218–27. doi: 10.1039/d4ra01765j (PMC11103566; doi:10.1039/d4ra01765j)
Supplement: RA-014-D4RA01765J-s001 [file RA-014-D4RA01765J-s001.pdf]

Electronic Supplementary Material (ESI) for RSC Advances.
This journal is © The Royal Society of Chemistry 2024

**Synthesis, biological evaluation, and molecular docking of Novel Ferulic Acid Derivatives Containing the 1,3,4-Oxadiazole thioether and trifluoromethyl pyrimidine skeleton**

**Jiansong An[1], Nianjuan Pan[1], Chunyi Liu[1], Haijiang Chen[1], Qiang Fei[1], Xiuhai Gan[2] and Wenneng Wu[1,*]**

1. School of Food Science and Engineering, Guiyang University, Guiyang 550005, China

2. National Key Laboratory of Green Pesticide, Key Laboratory of Green Pesticide and Agricultural Bioengineering, Ministry of Education, Guizhou University, Guiyang 550025, China

*Correspondence: wuwenneng123@126.com (Wenneng Wu)

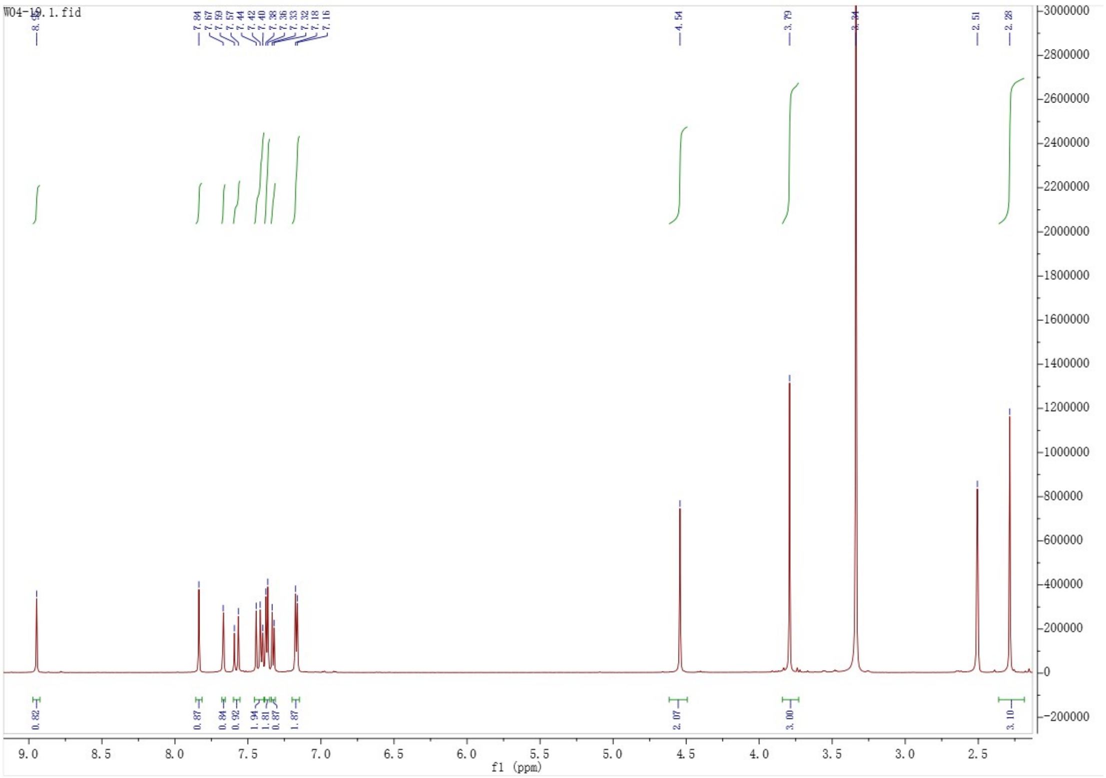


Figure S1. $^{1}$H NMR spectrum of compound **6a**

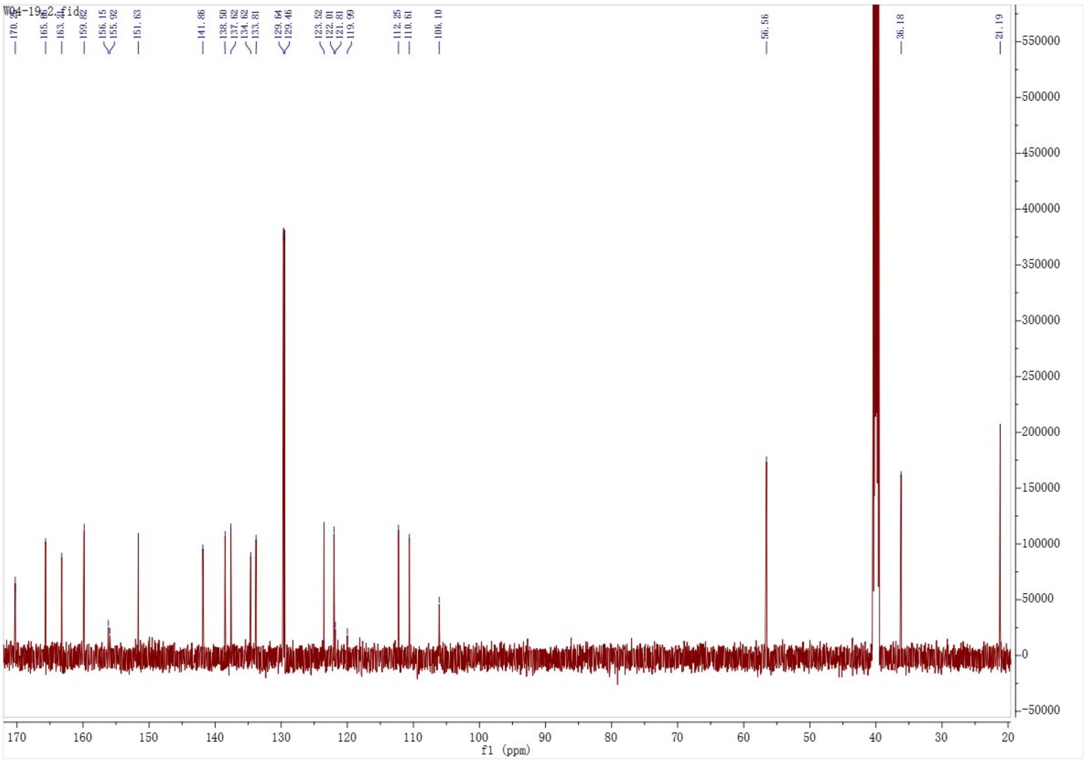


Figure S2. $^{13}$C NMR spectrum of compound **6a**

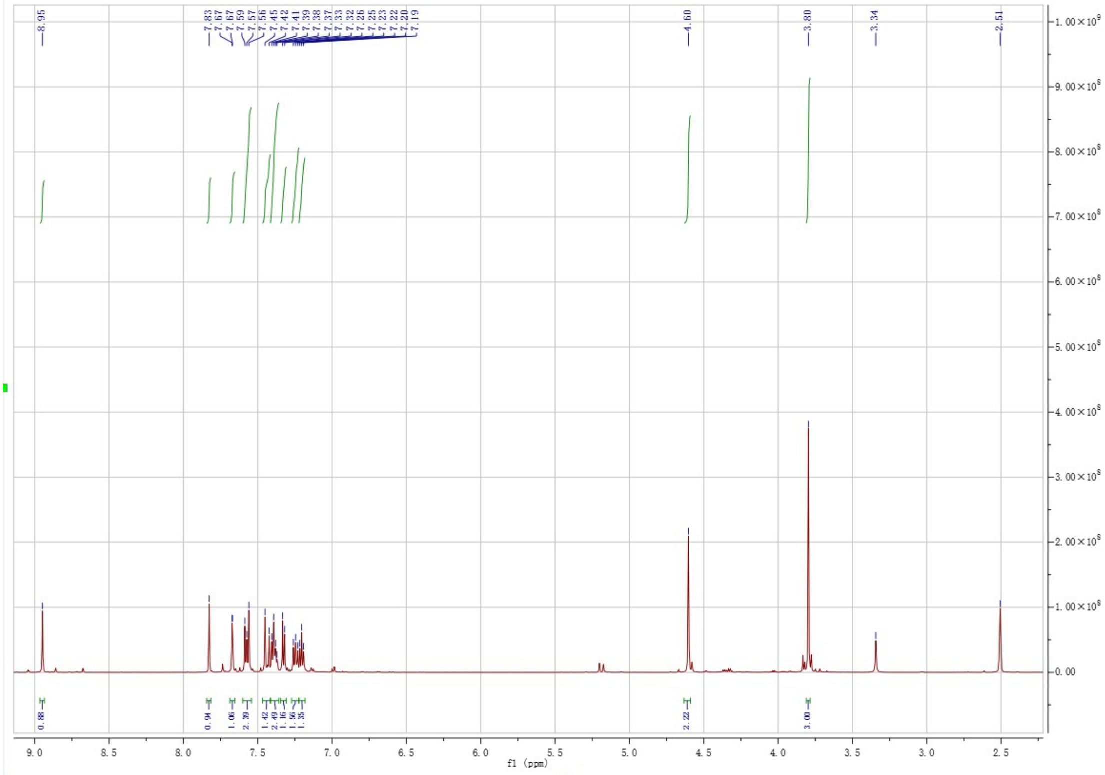

Figure S3. $^{1}$H NMR spectrum of compound **6b**

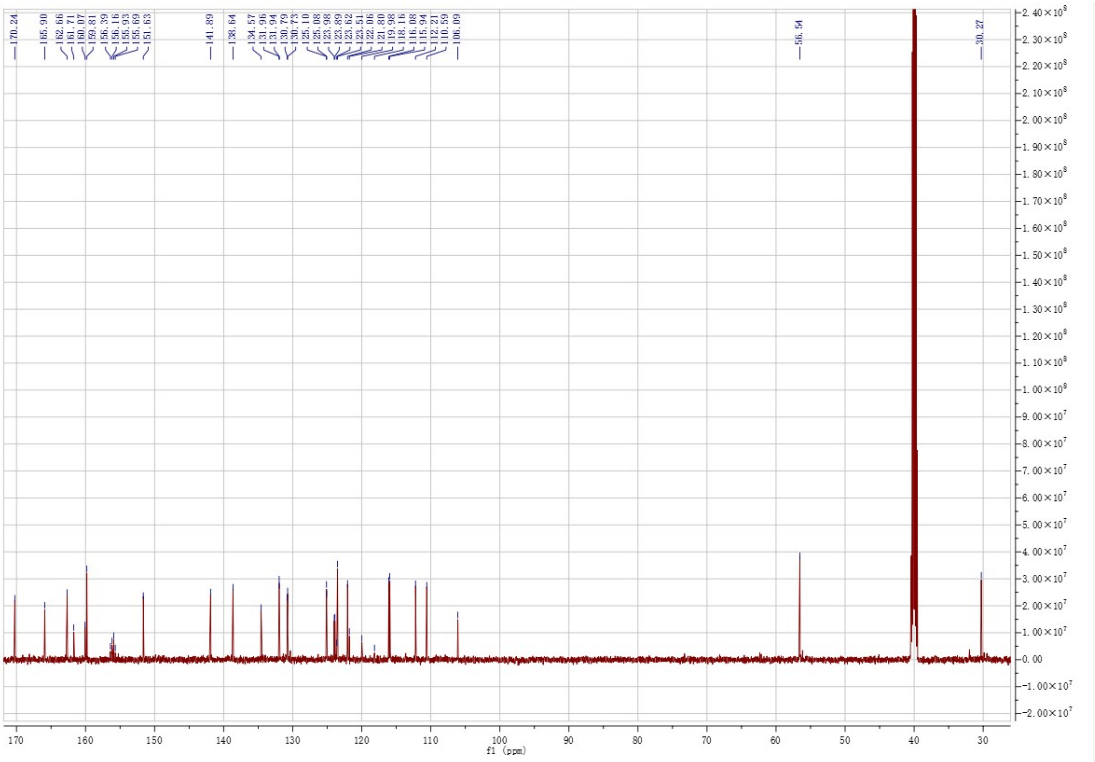

Figure S4. $^{13}$C NMR spectrum of compound **6b**

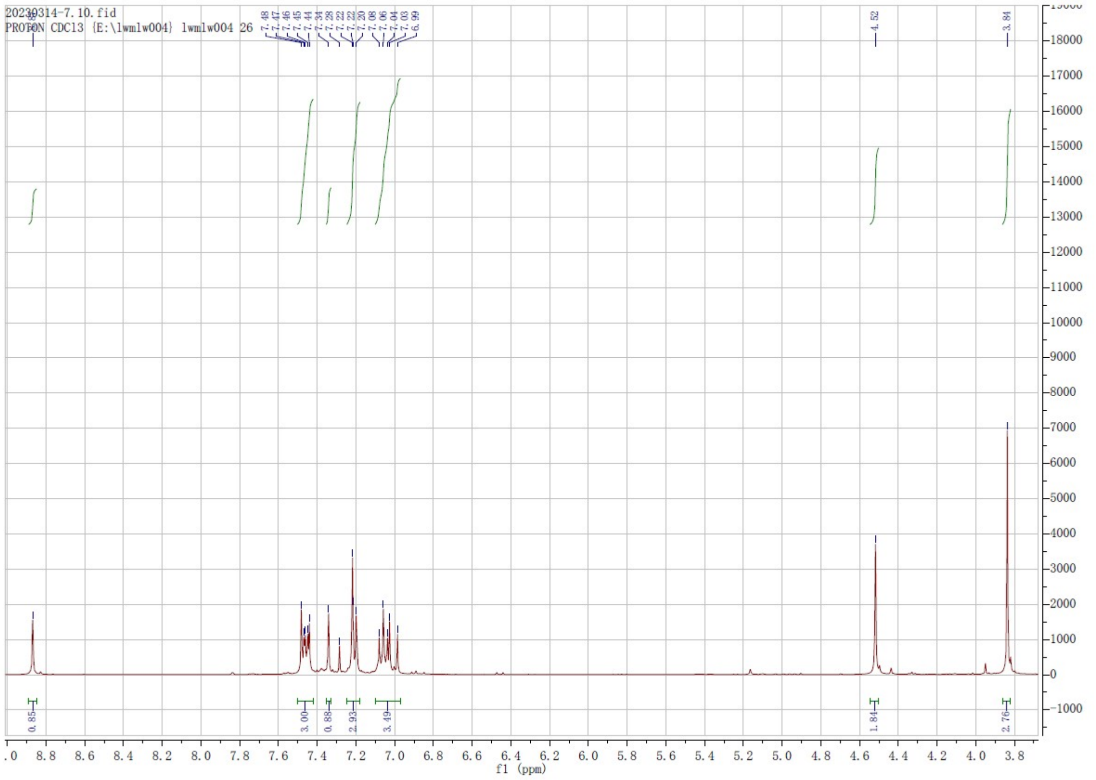


Figure S5. $^{1}$H NMR spectrum of compound **6c**

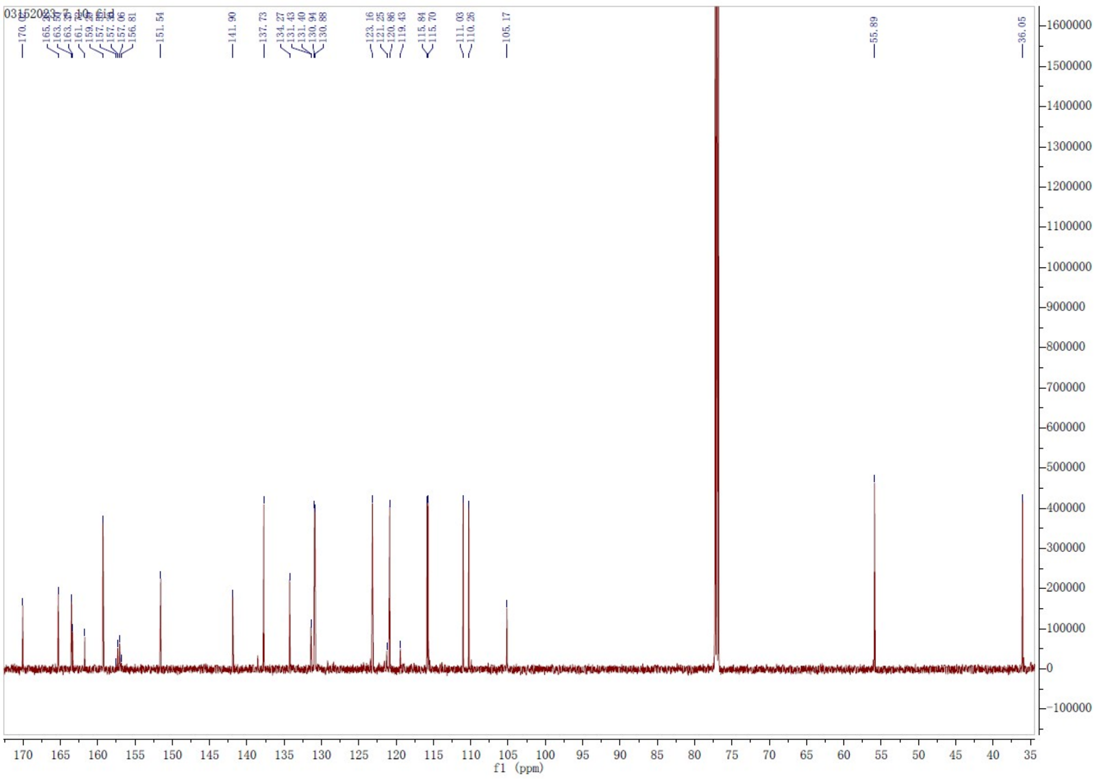


Figure S6. $^{13}$C NMR spectrum of compound **6c**

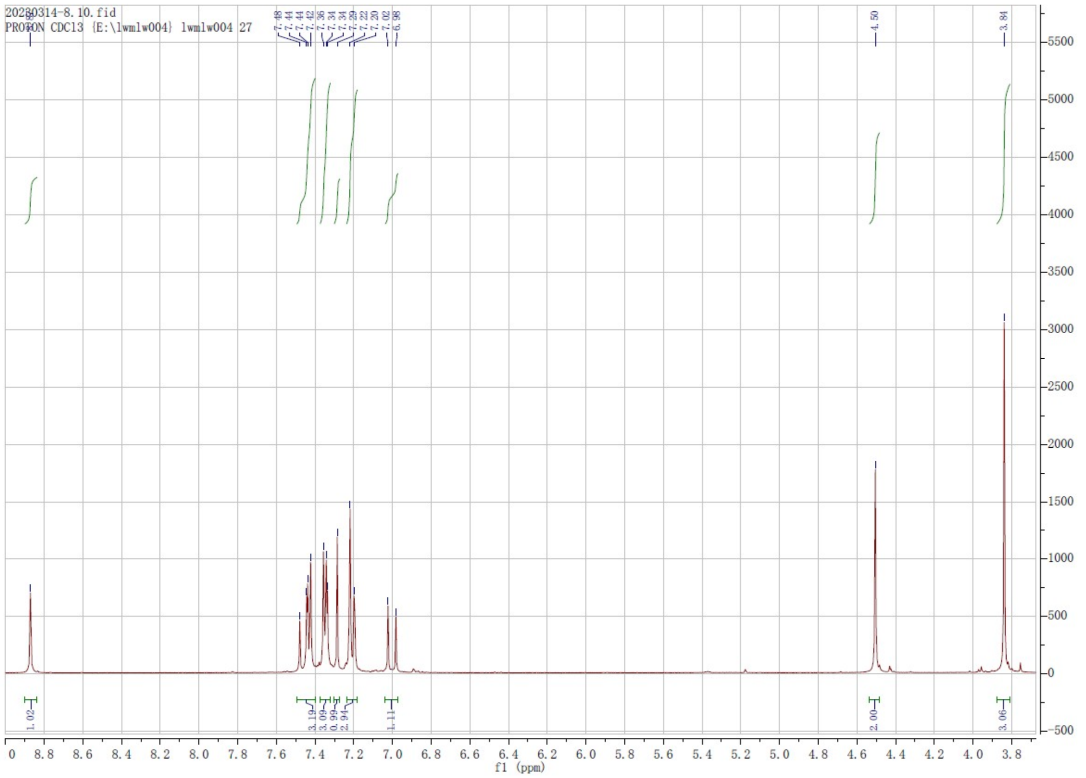

Figure S7. $^{1}$H NMR spectrum of compound **6d**

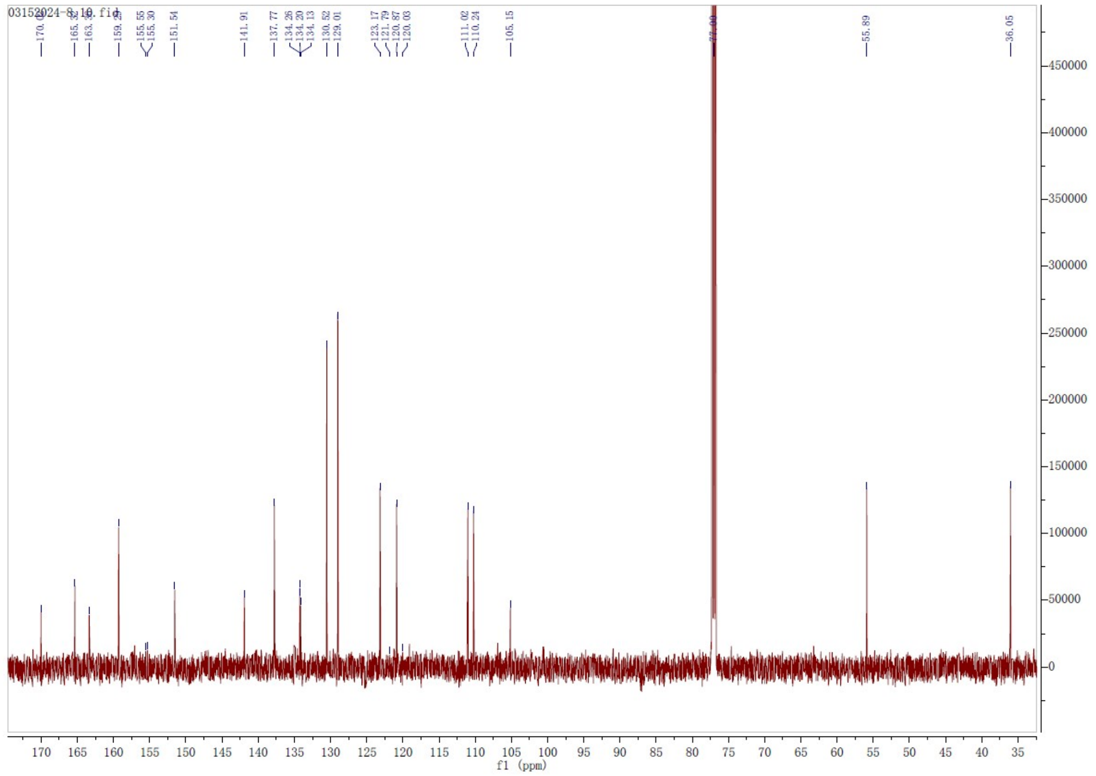

Figure S8. $^{13}$C NMR spectrum of compound **6d**

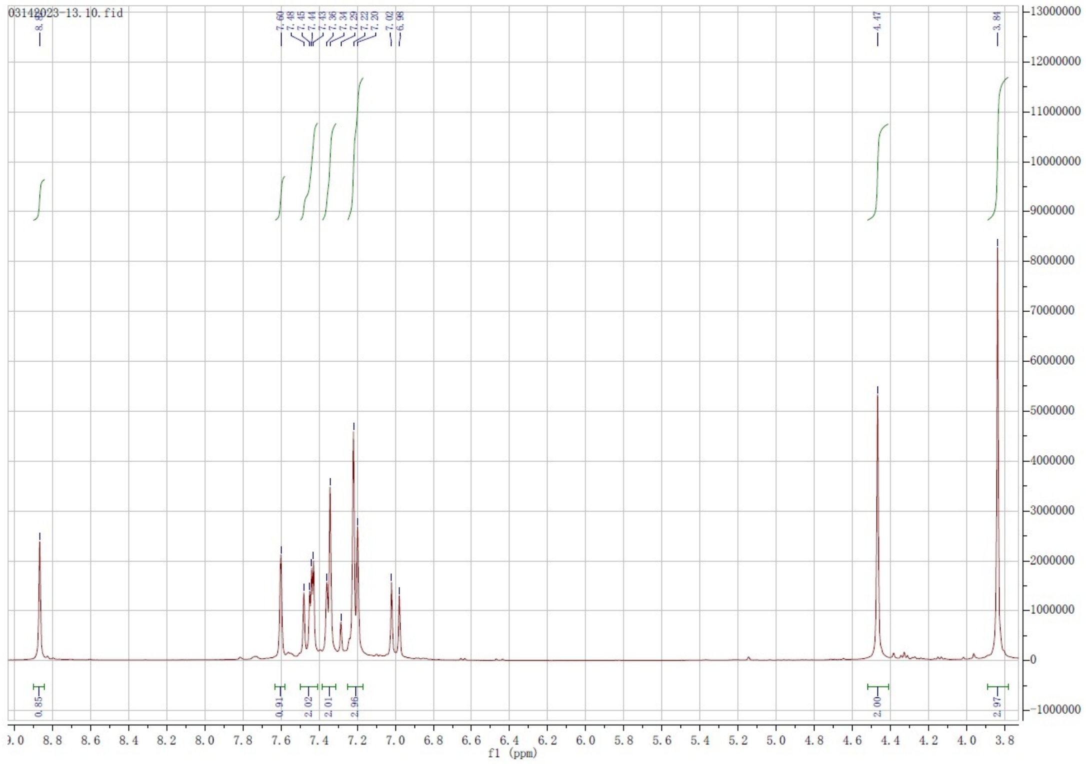

Figure S9. $^{1}$H NMR spectrum of compound **6e**

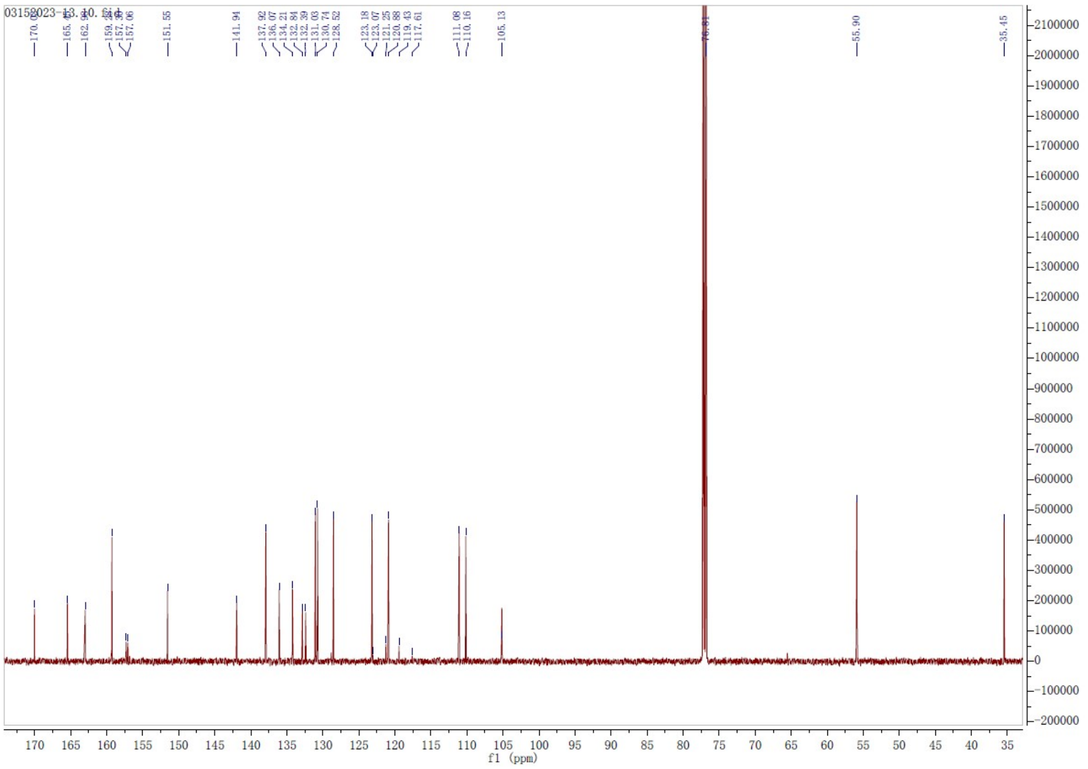

Figure S10. $^{13}$C NMR spectrum of compound **6c**

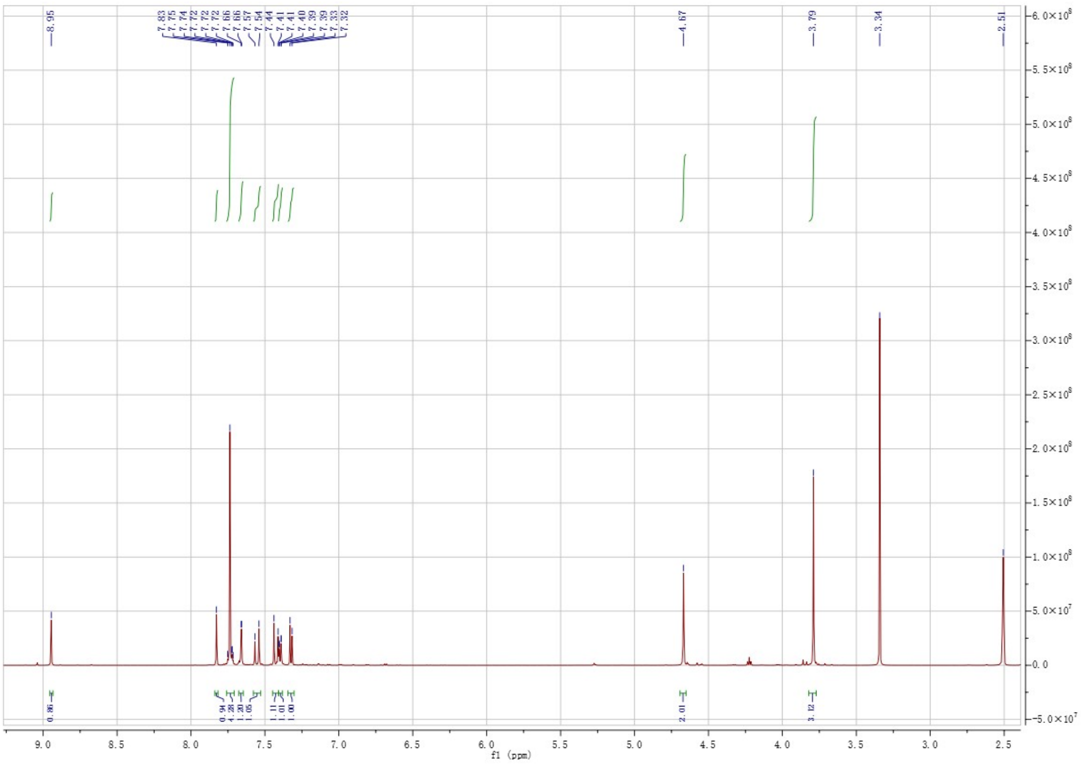


Figure S11. $^{1}$H NMR spectrum of compound **6f**

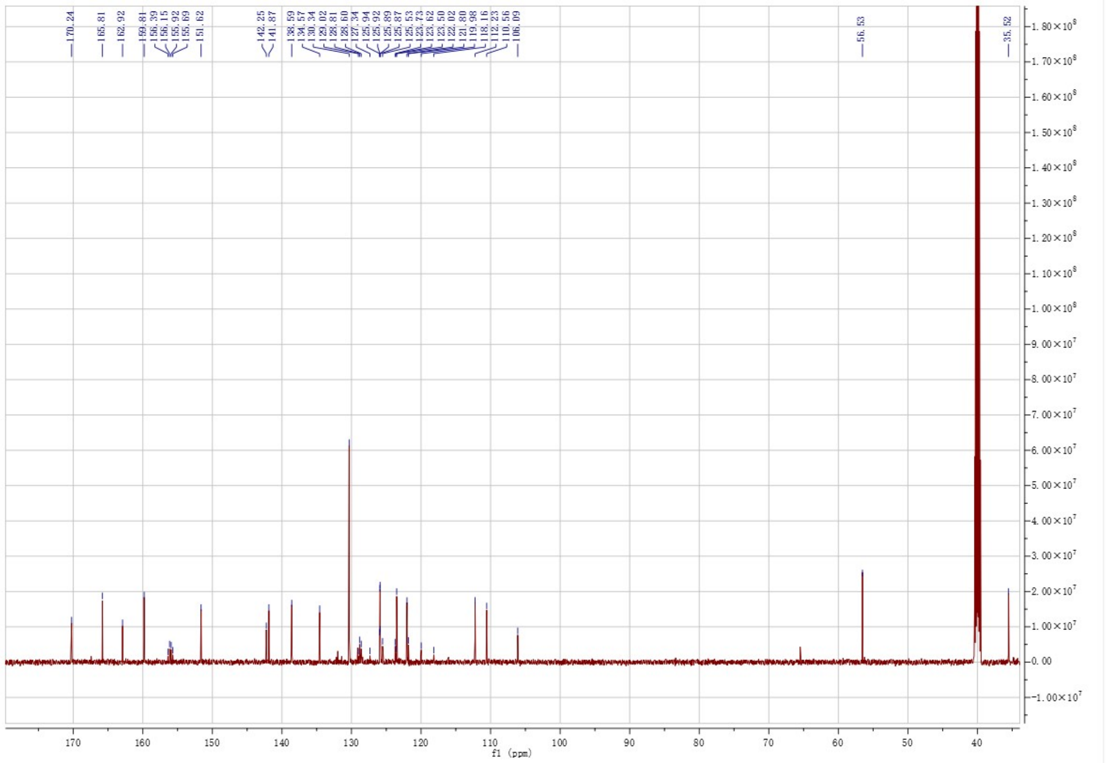


Figure S12. $^{13}$C NMR spectrum of compound **6f**

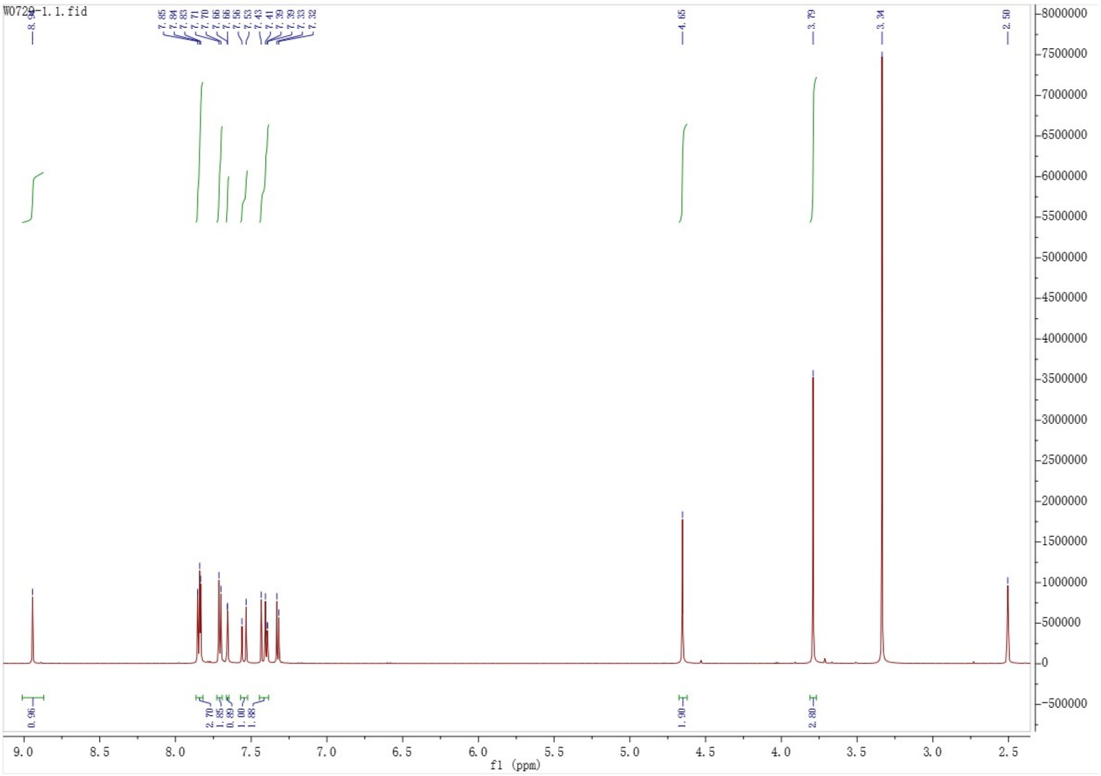

Figure S13. $^{1}H$ NMR spectrum of compound **6g**

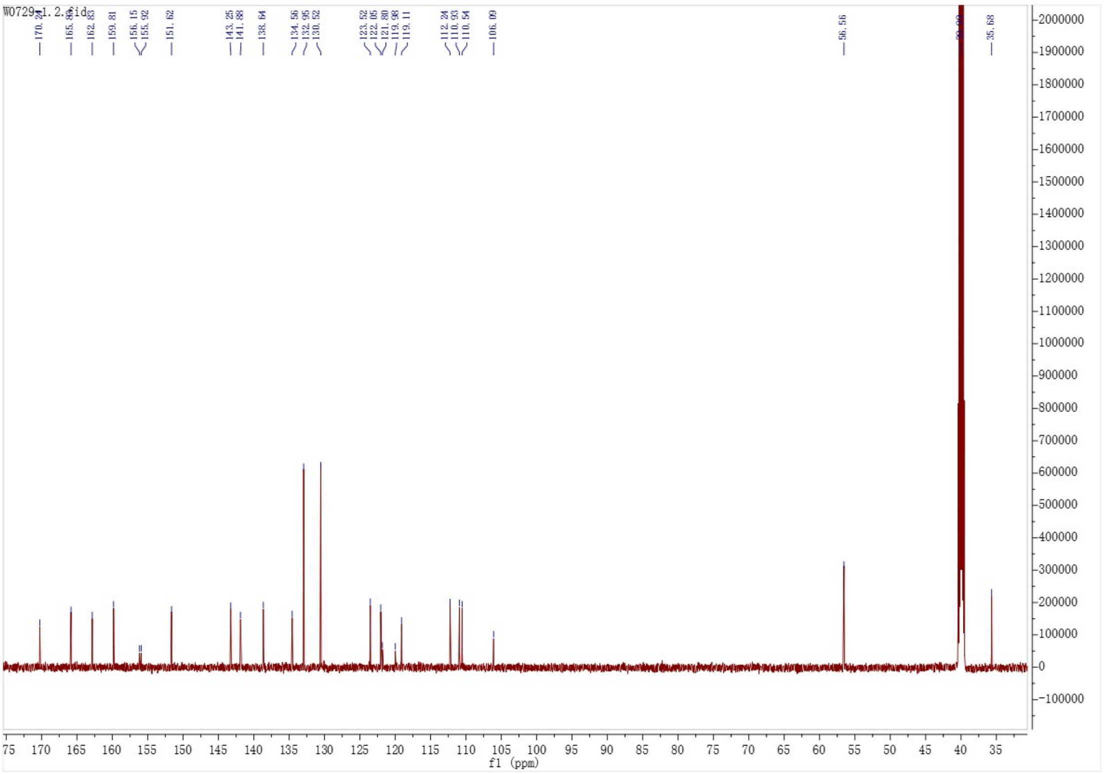

Figure S14. $^{13}C$ NMR spectrum of compound **6g**

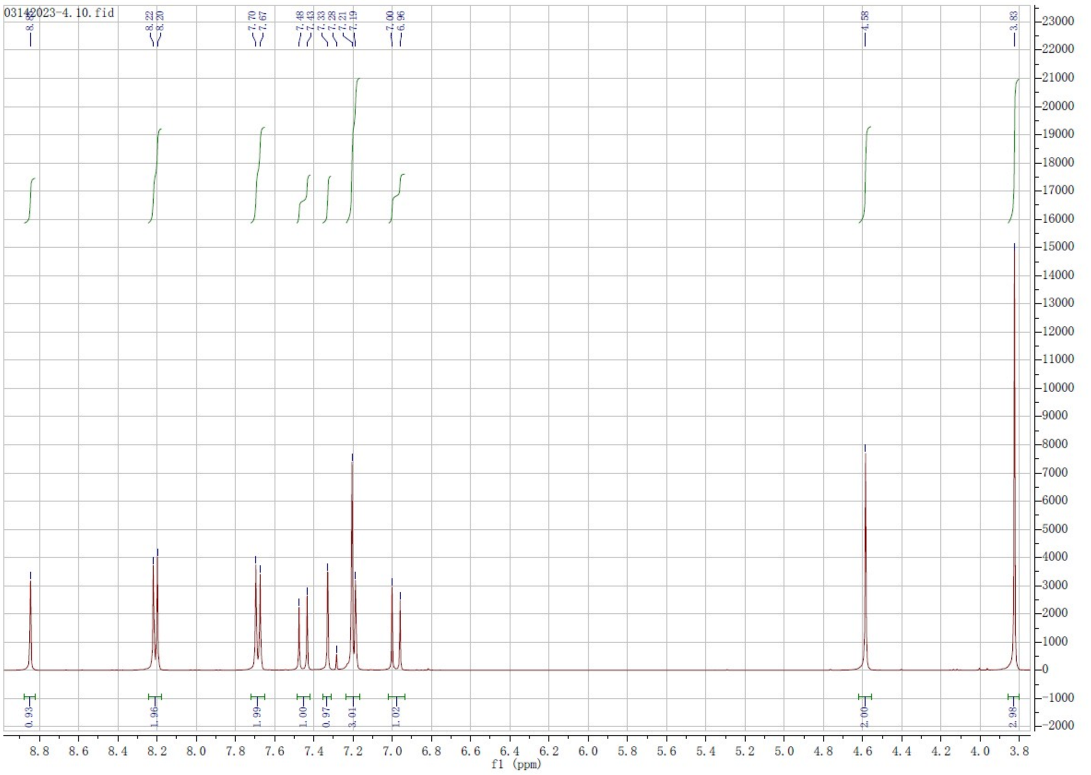


Figure S15. $^{1}$H NMR spectrum of compound **6h**

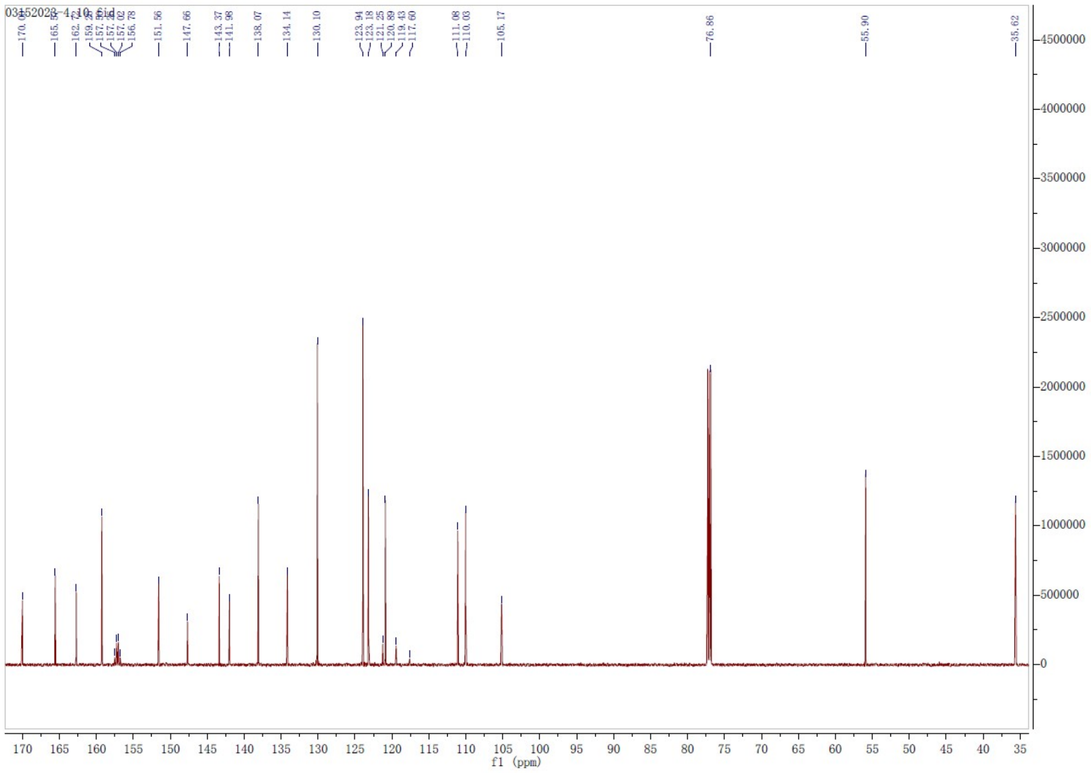


Figure S16. $^{13}$C NMR spectrum of compound **6h**

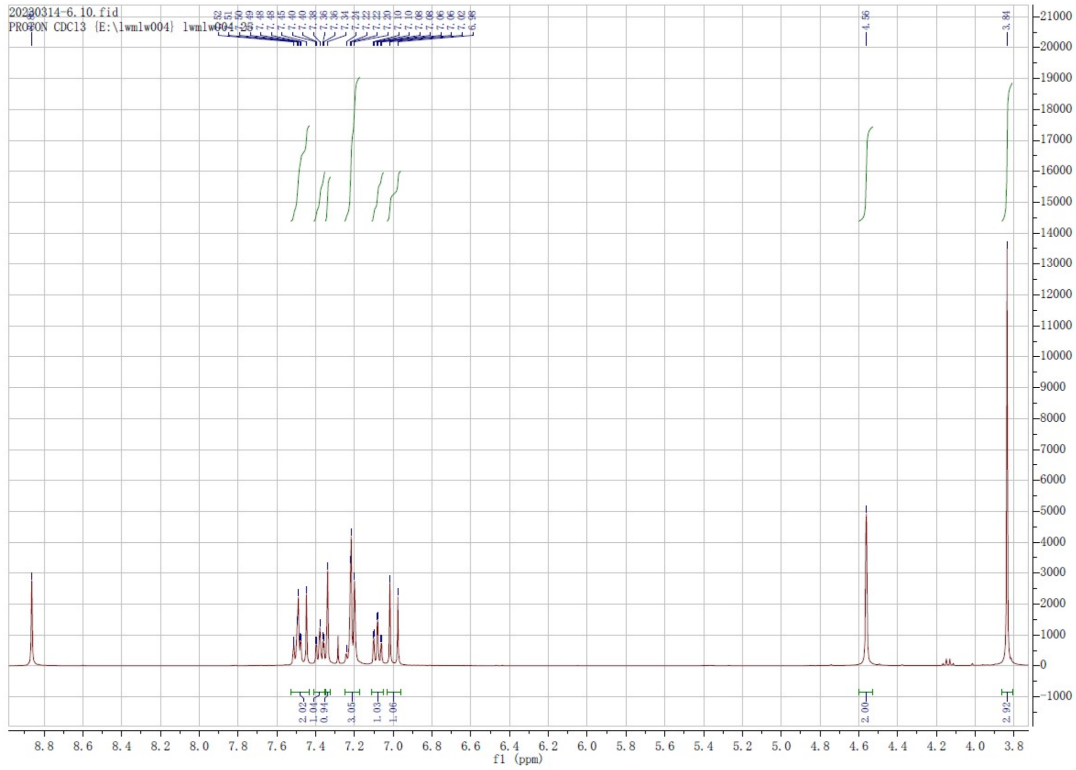

Figure S17. $^{1}H$ NMR spectrum of compound **6i**

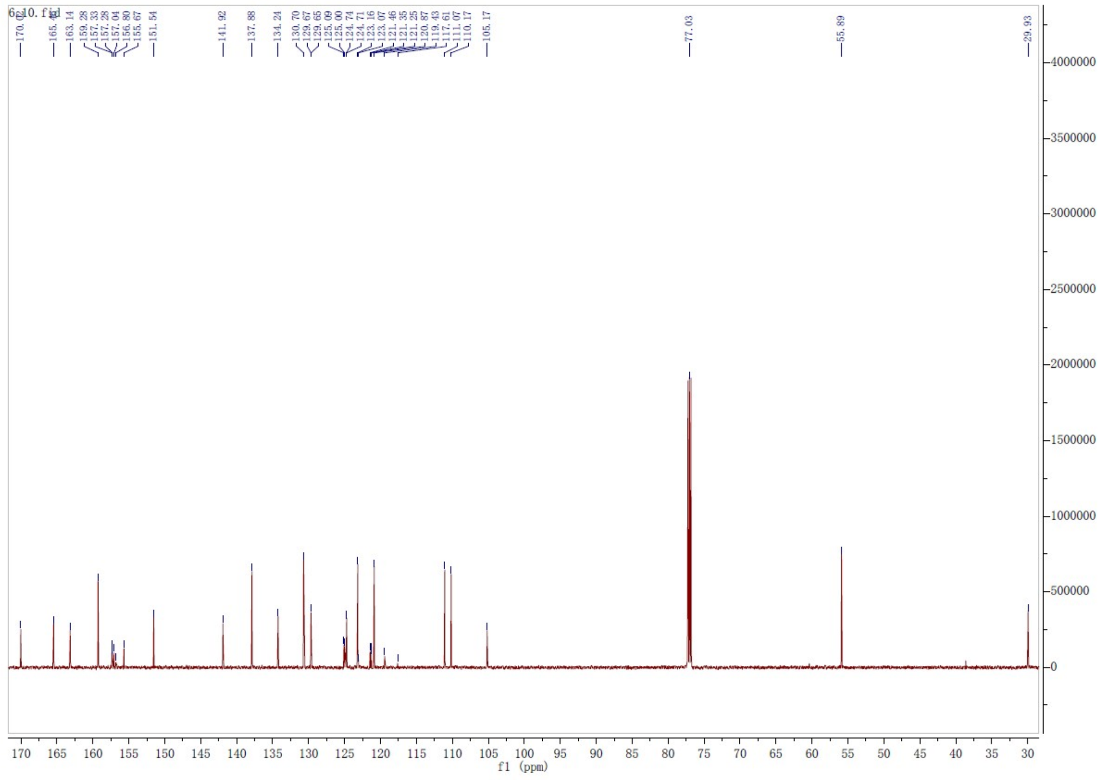

Figure S18. $^{13}C$ NMR spectrum of compound **6i**

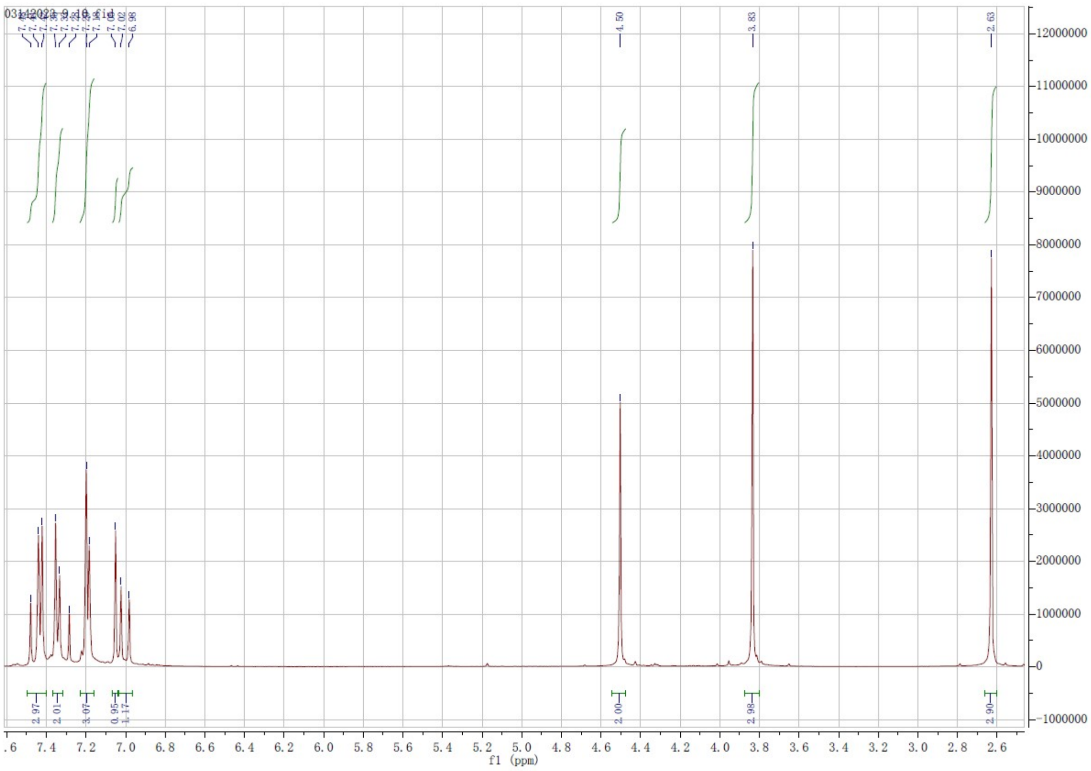

Figure S19. $^{1}$H NMR spectrum of compound **6j**

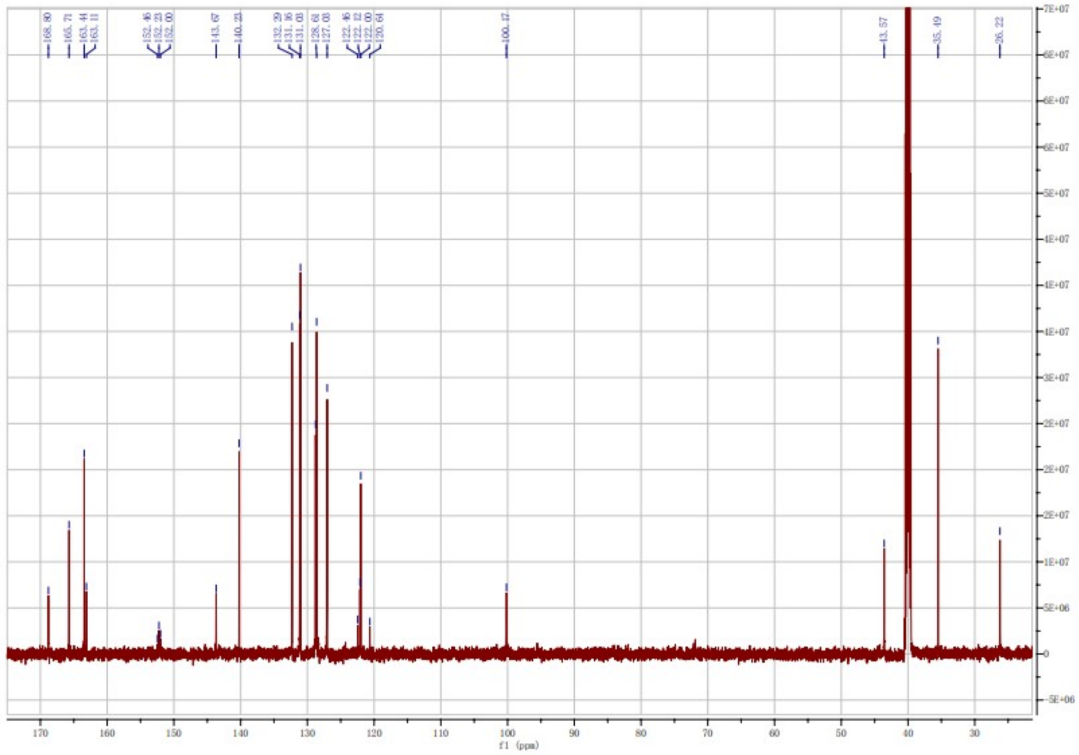

Figure S20. $^{13}$C NMR spectrum of compound **6j**

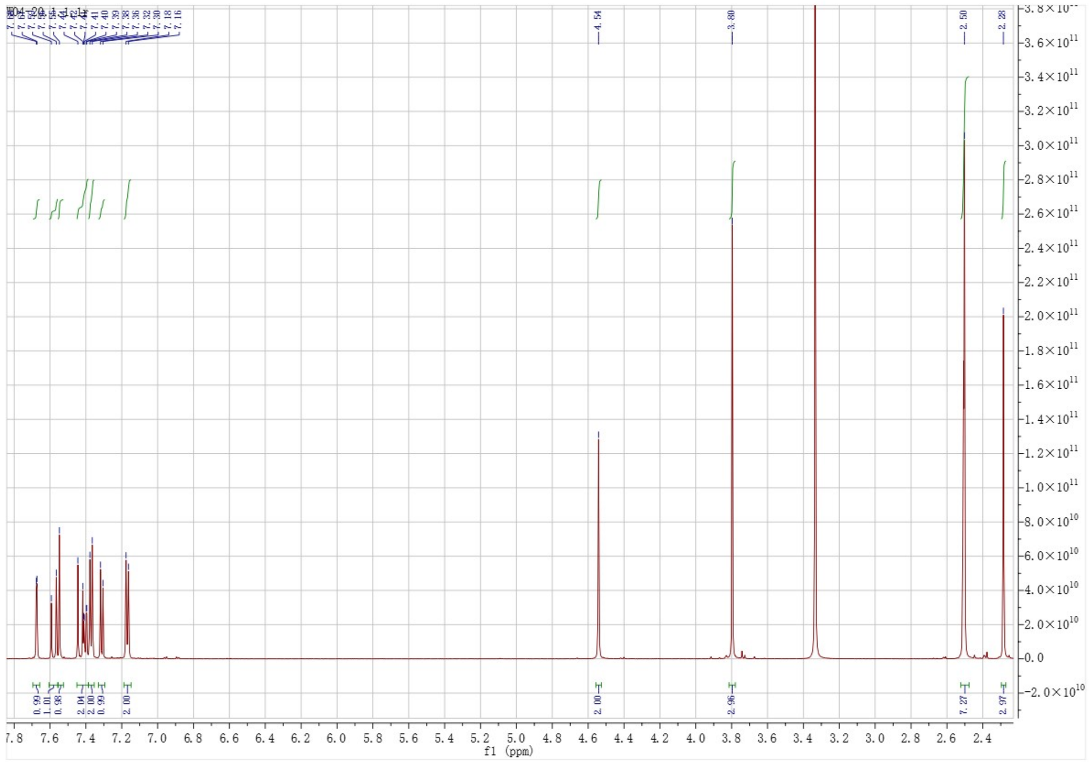

Figure S21. $^{1}H$ NMR spectrum of compound **6k**

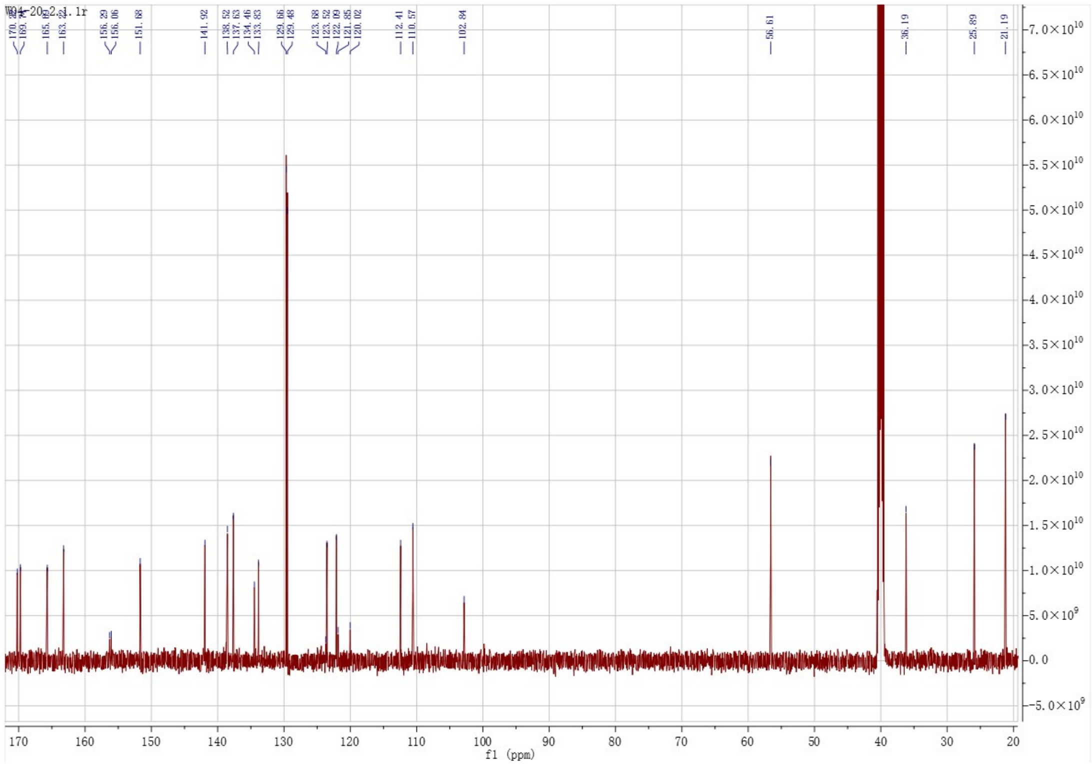

Figure S22. $^{13}C$ NMR spectrum of compound **6k**

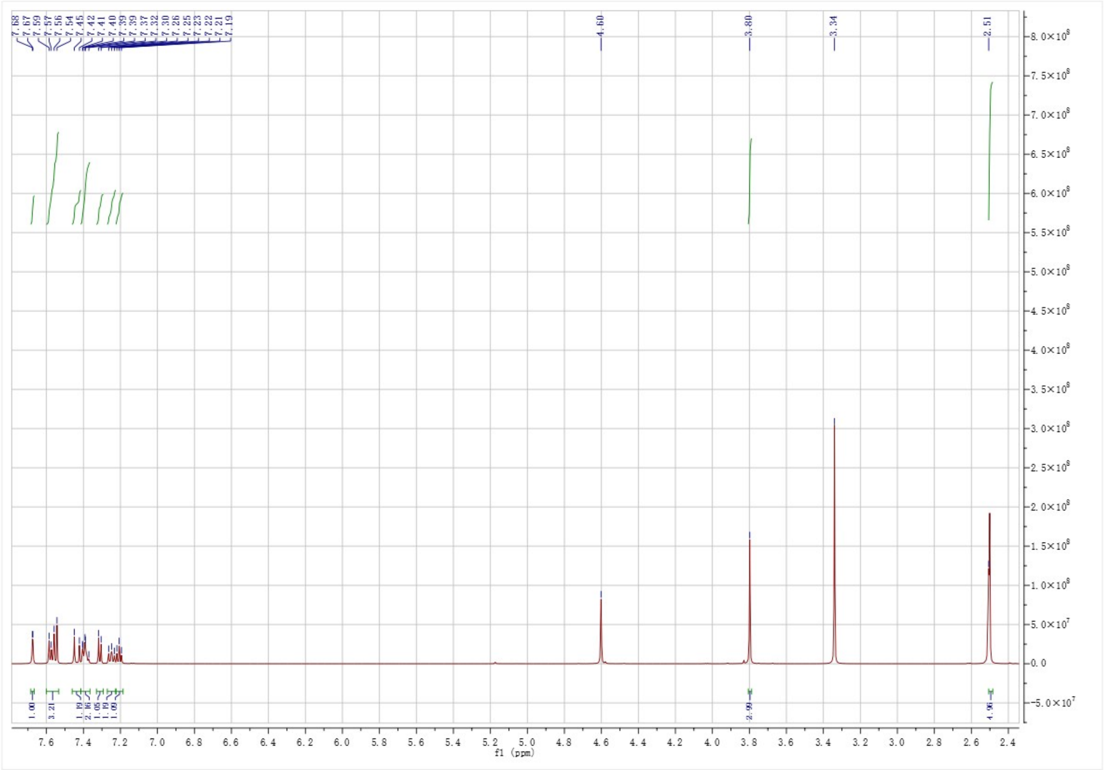

Figure S23. $^{1}$H NMR spectrum of compound **6l**

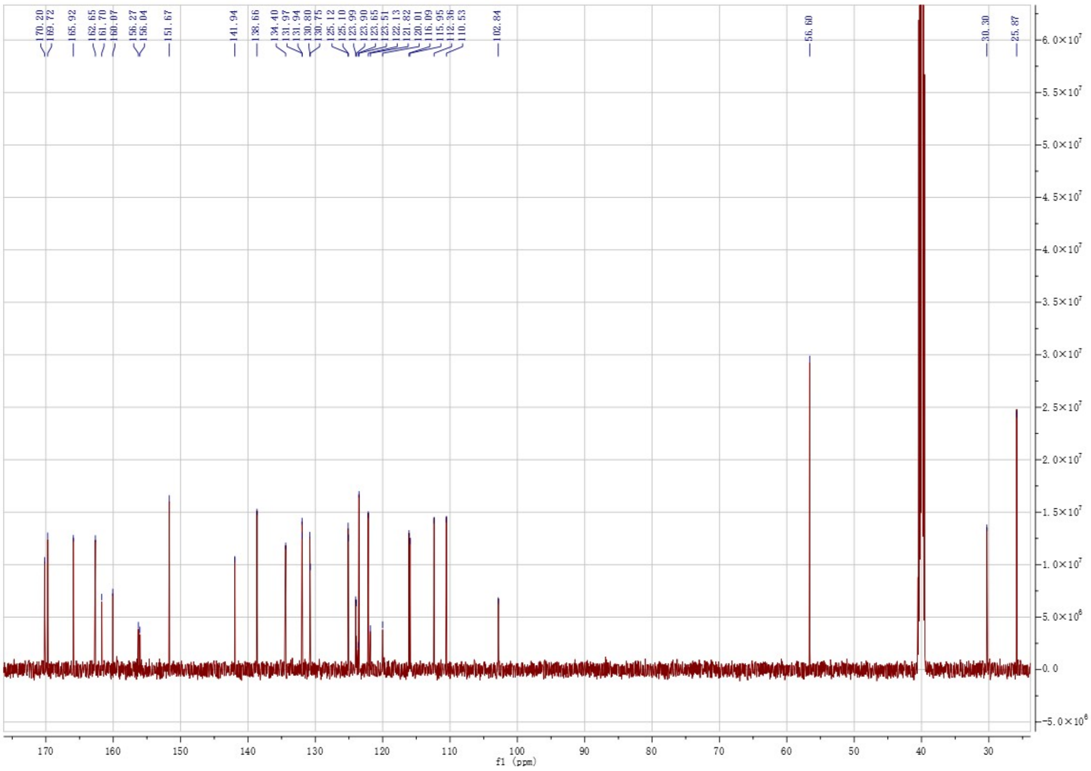

Figure S24. $^{13}$C NMR spectrum of compound **6l**

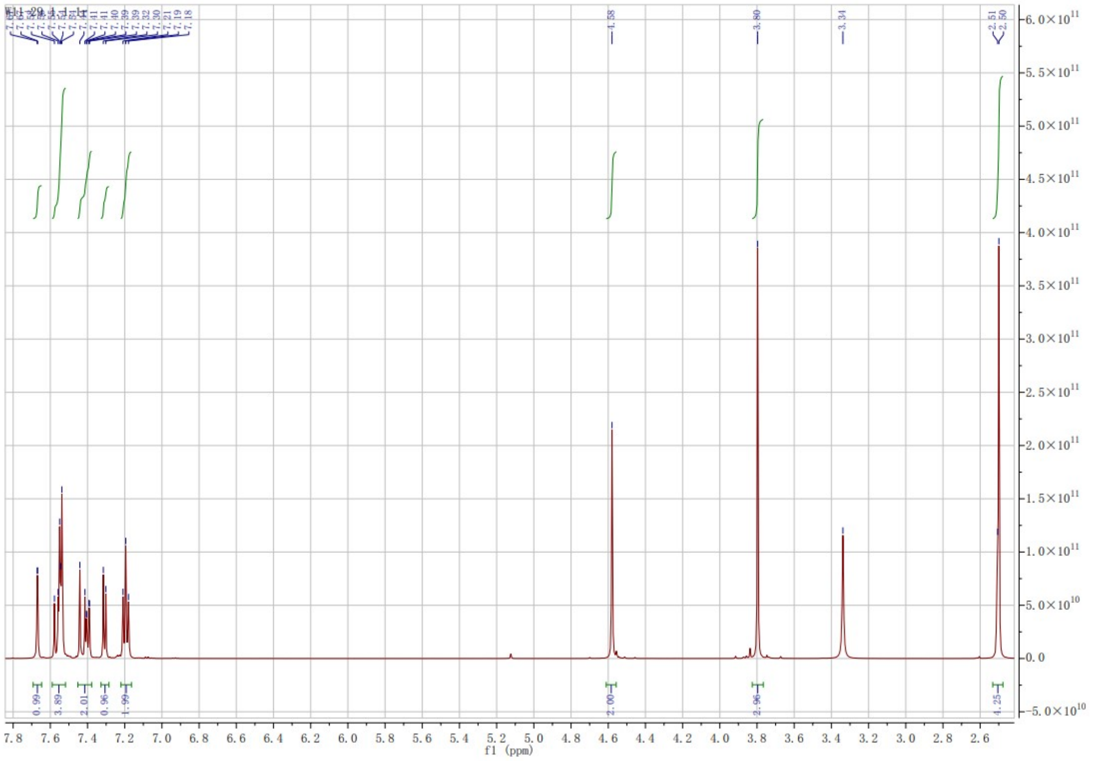

Figure S25. $^{1}$H NMR spectrum of compound **6m**

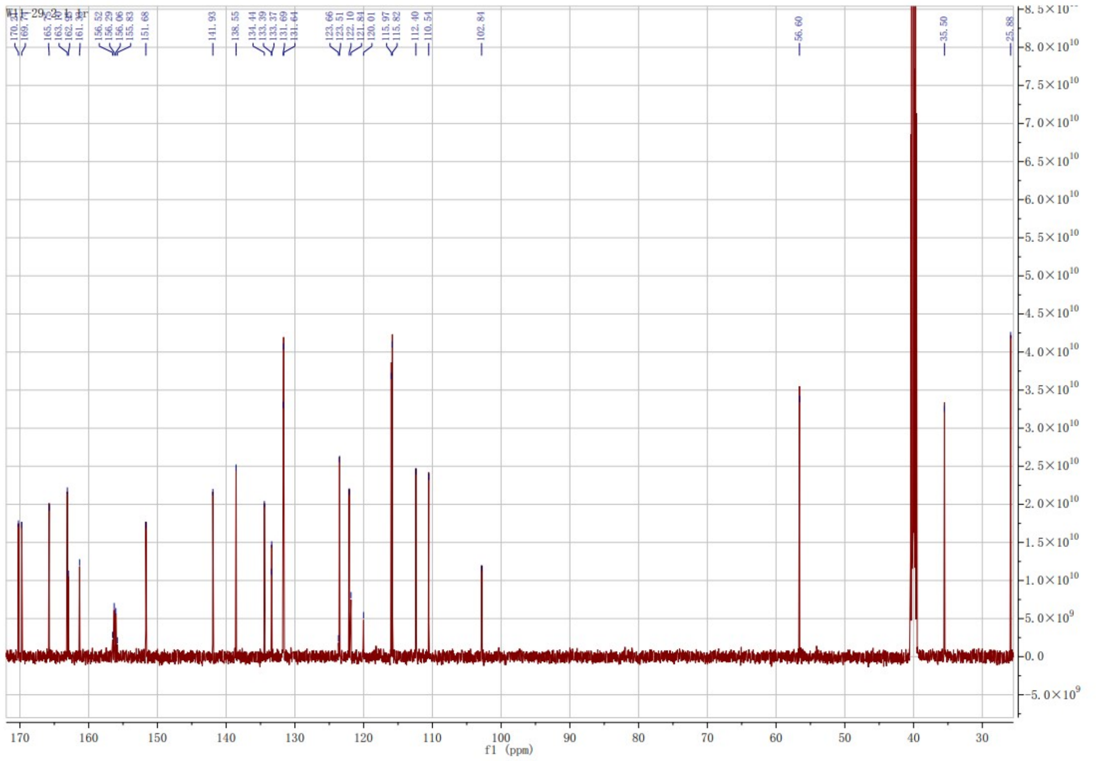

Figure S26. $^{13}$C NMR spectrum of compound **6m**

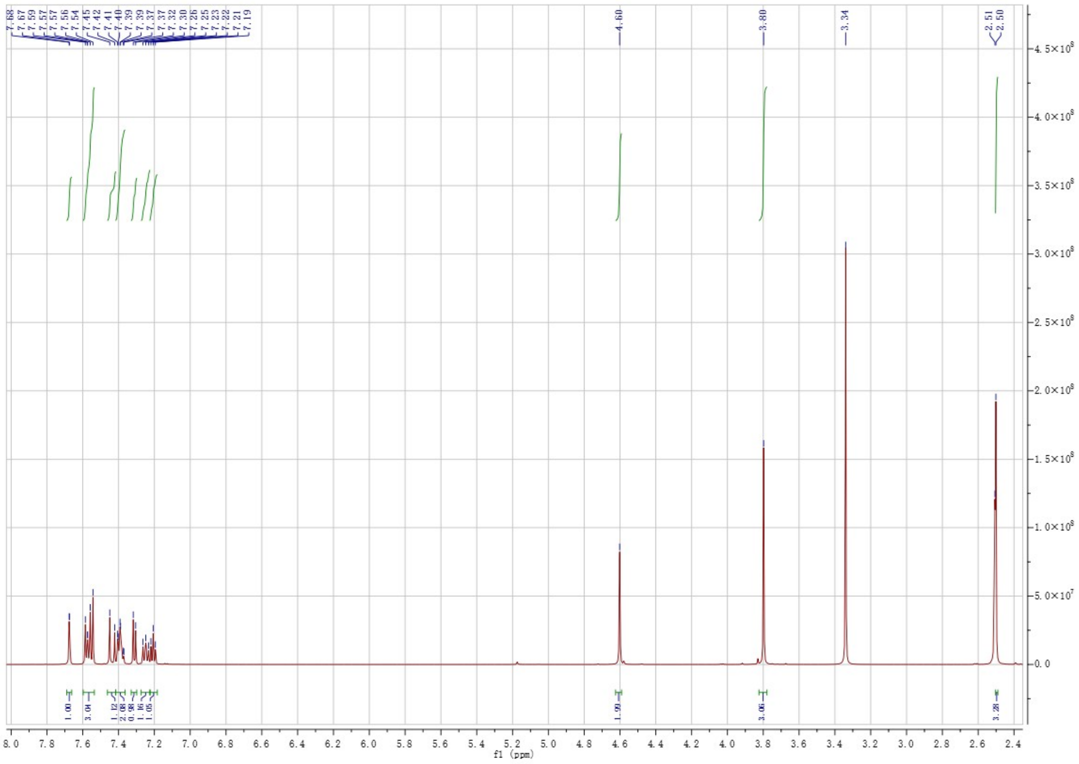


Figure S27. $^{1}$H NMR spectrum of compound **6n**

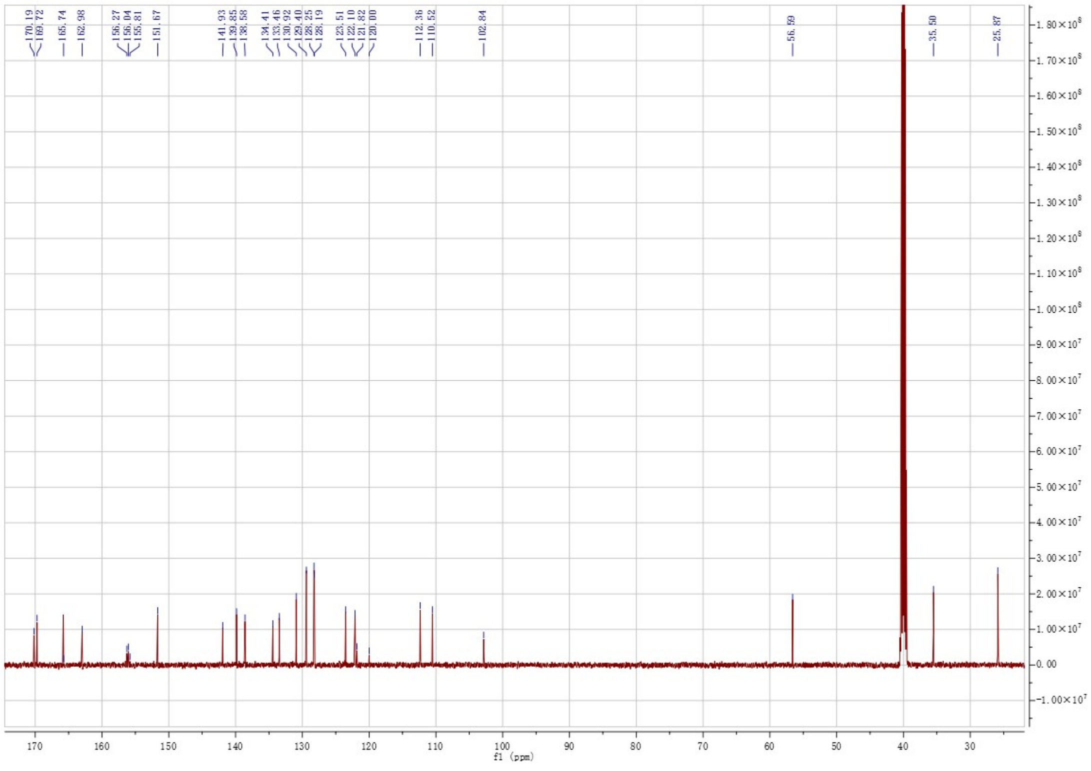


Figure S28. $^{13}$C NMR spectrum of compound **6n**

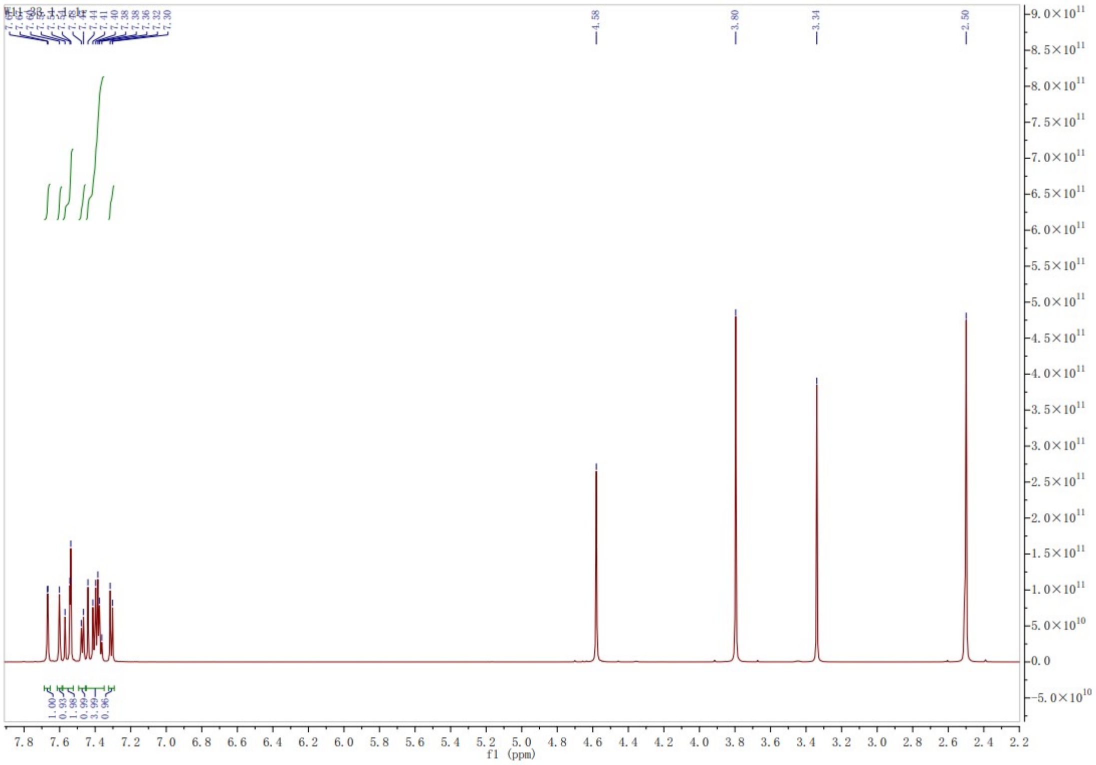

Figure S29. $^{1}$H NMR spectrum of compound **6o**

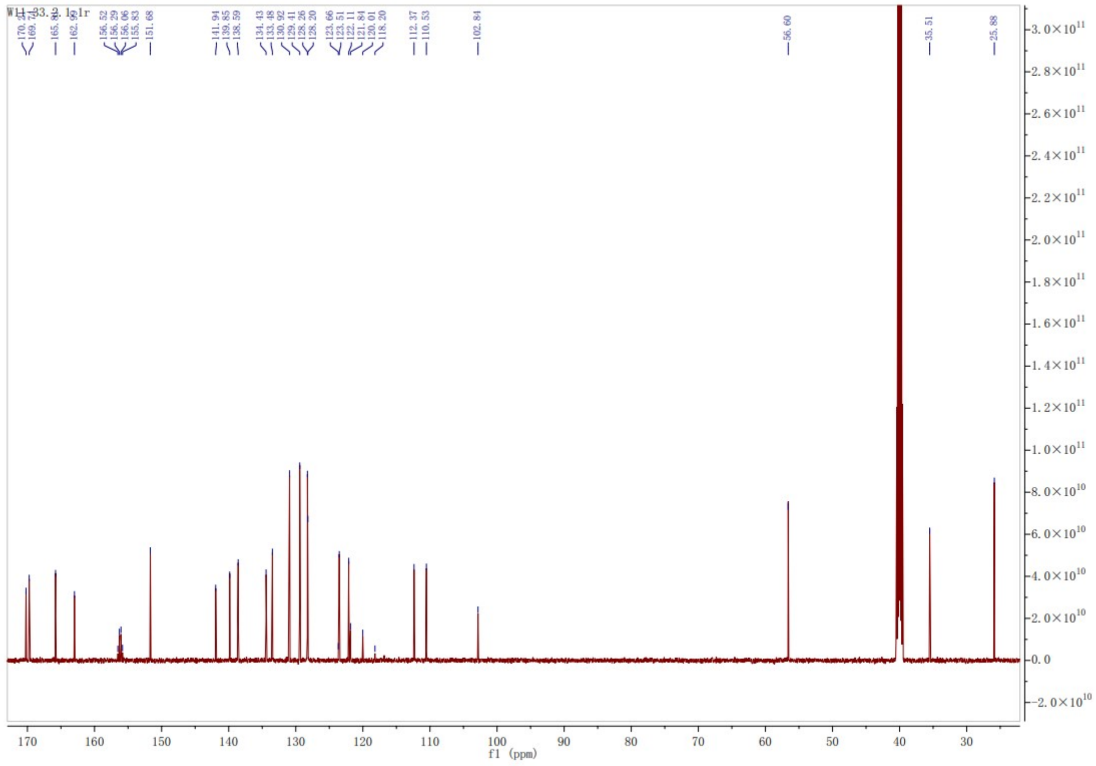

Figure S30. $^{13}$C NMR spectrum of compound **6o**

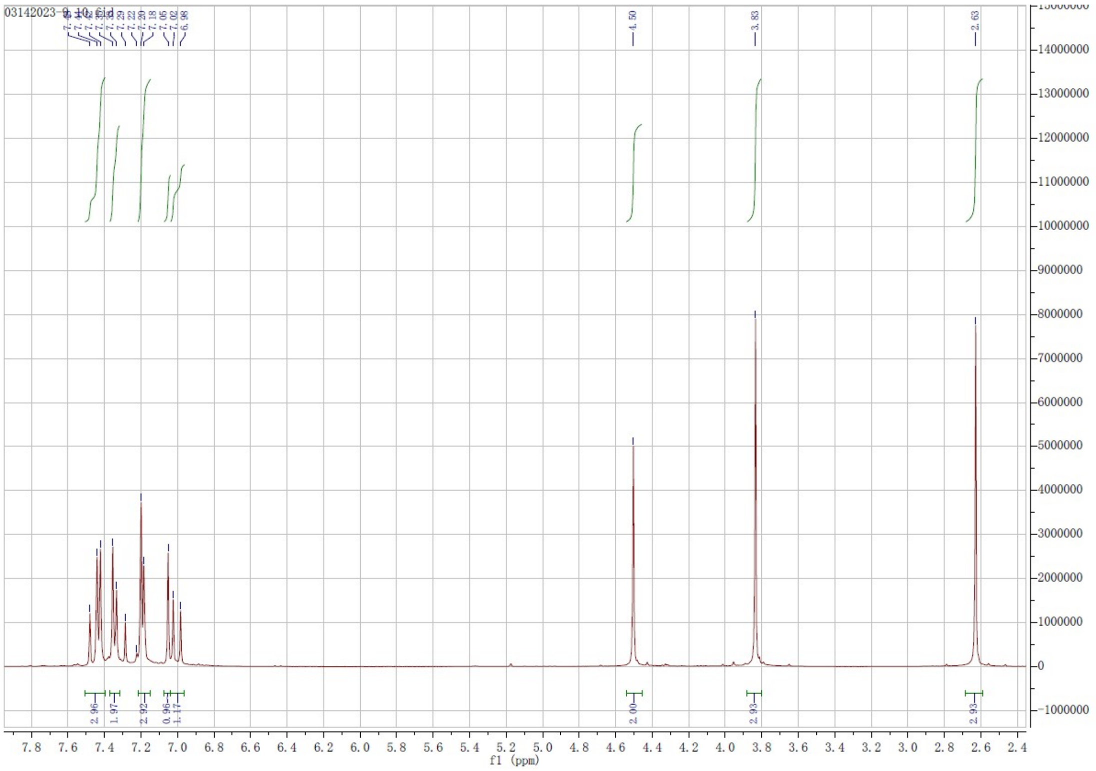


Figure S31. $^{1}$H NMR spectrum of compound **6p**

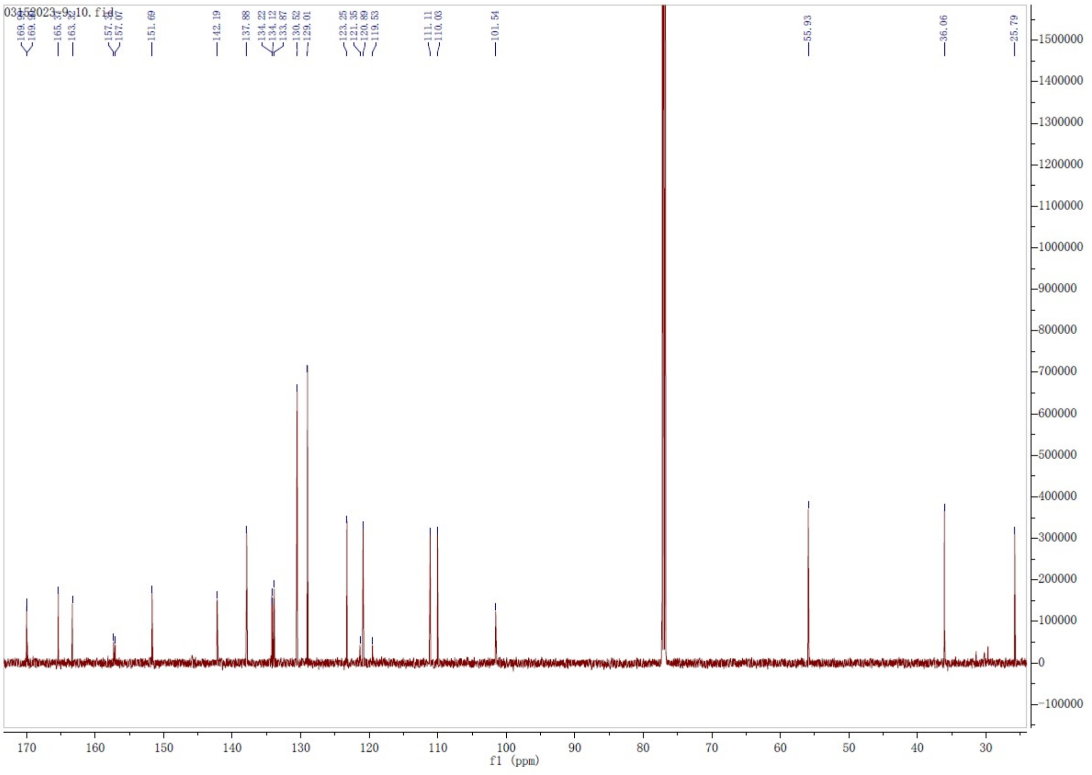


Figure S32. $^{13}$C NMR spectrum of compound **6p**

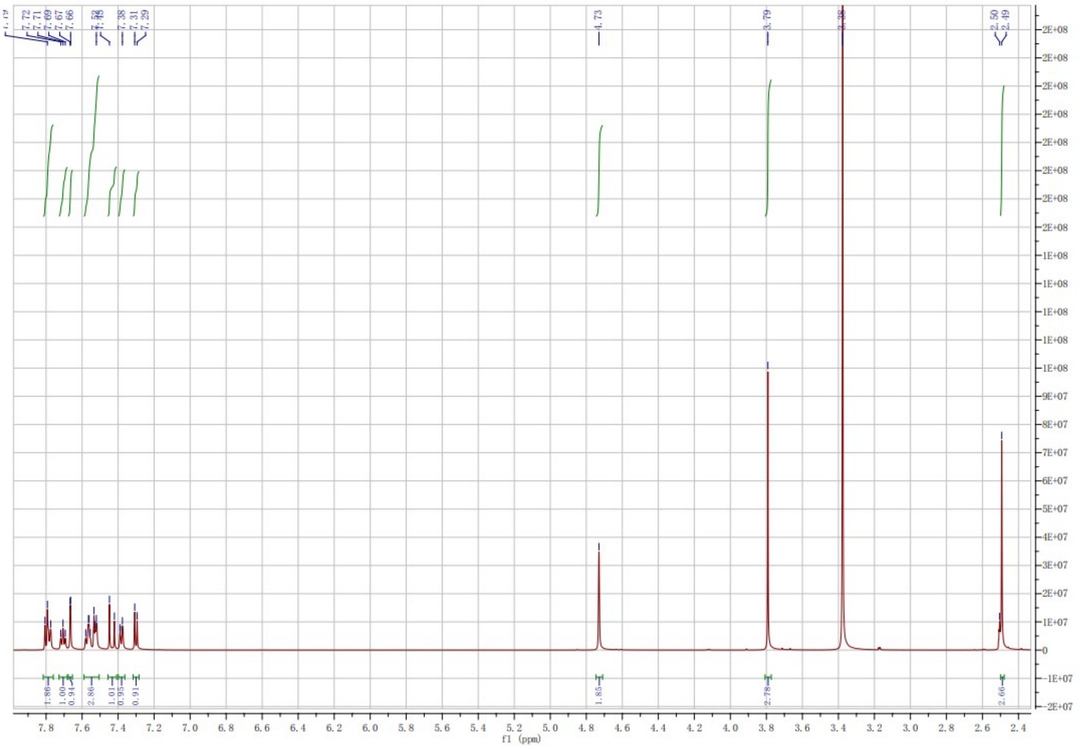

Figure S33. $^{1}H$ NMR spectrum of compound **6q**

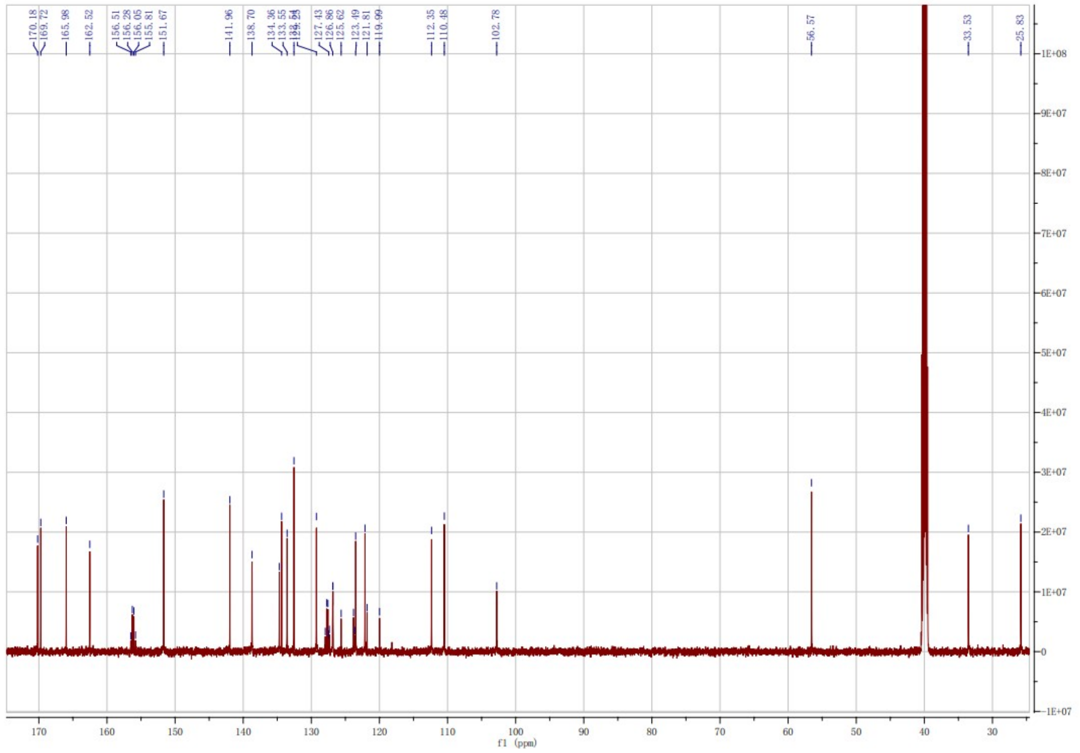

Figure S34. $^{13}C$ NMR spectrum of compound **6q**

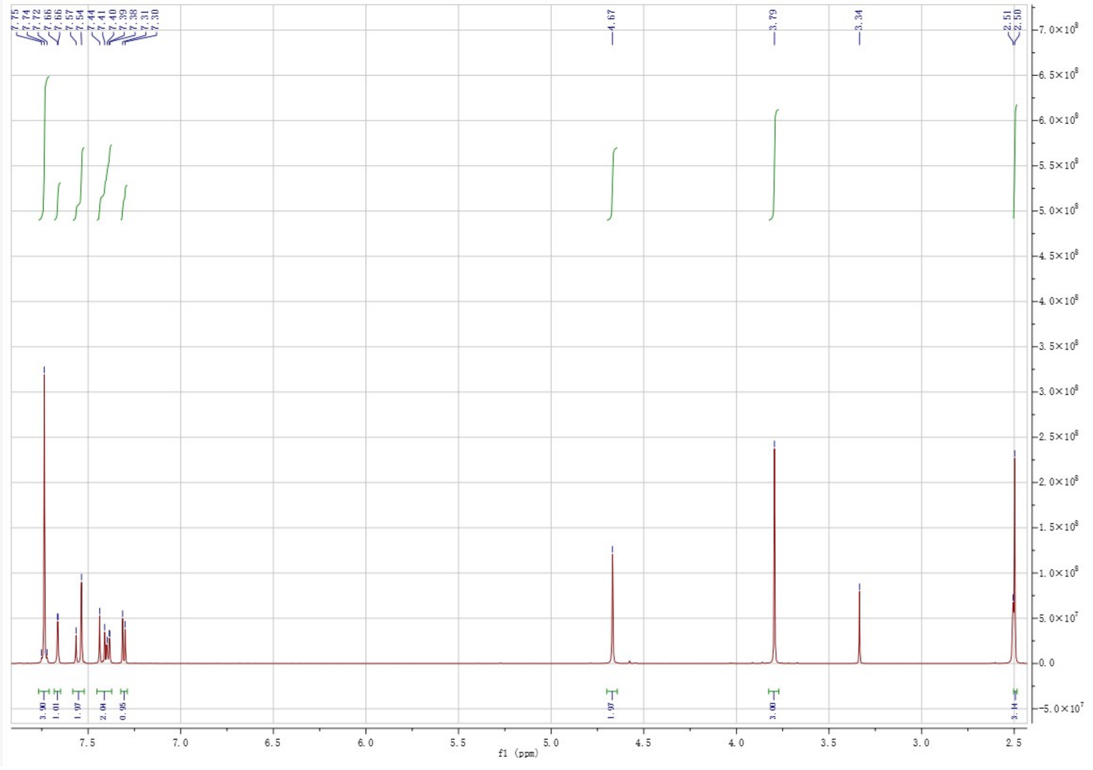


Figure S35. $^{1}$H NMR spectrum of compound **6r**

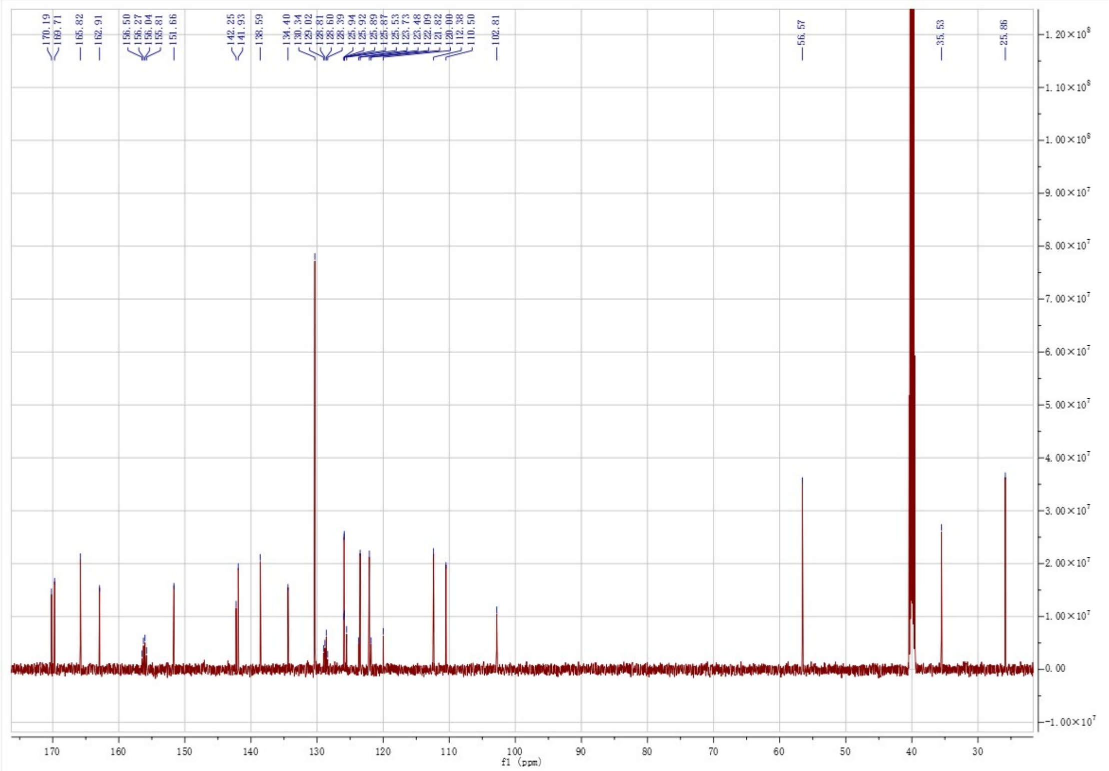


Figure S36. $^{13}$C NMR spectrum of compound **6r**

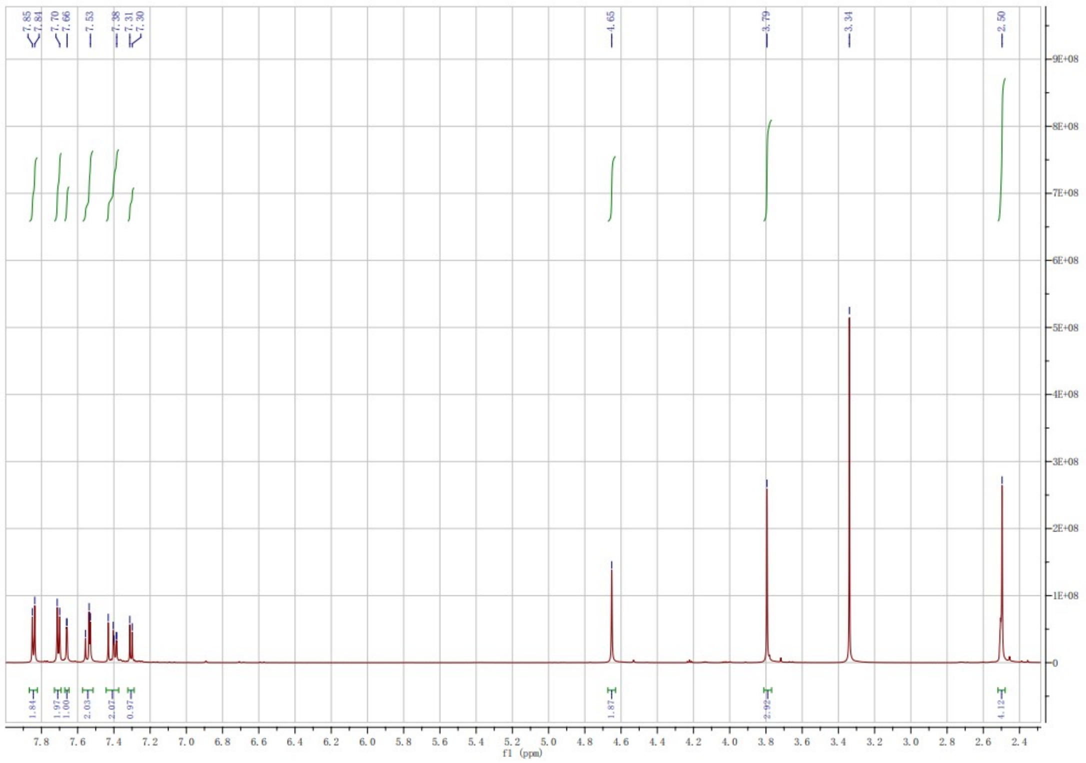

Figure S37. $^{1}H$ NMR spectrum of compound **6s**

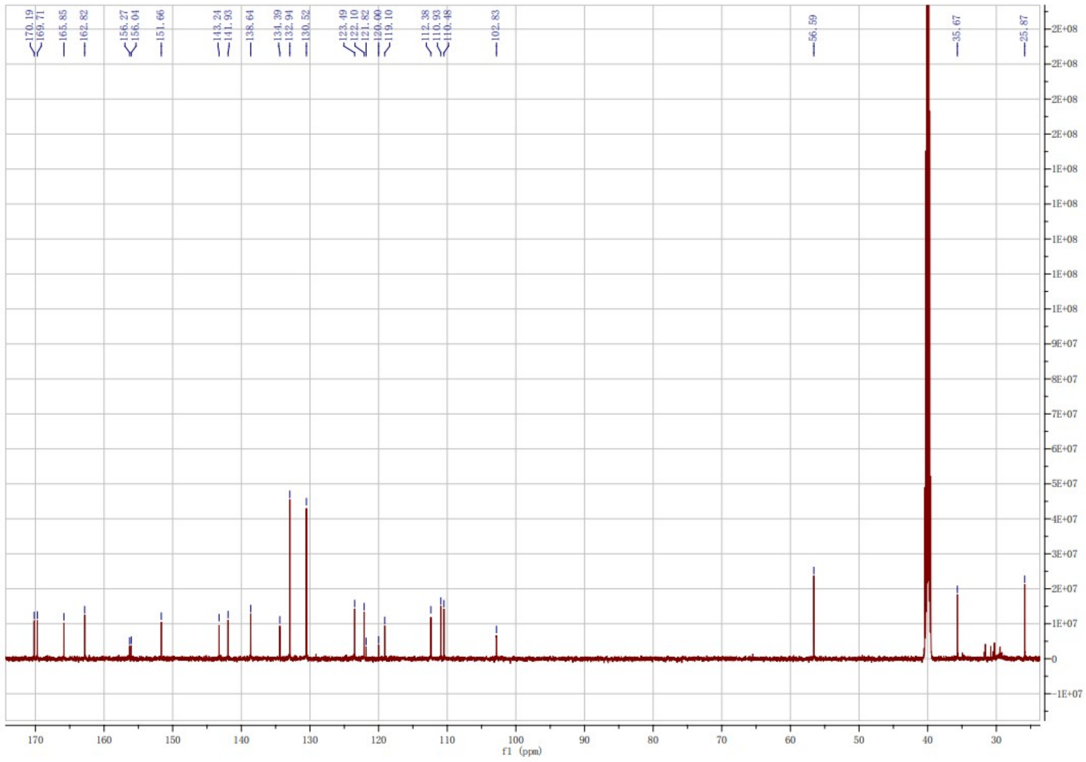

Figure S38. $^{13}C$ NMR spectrum of compound **6s**

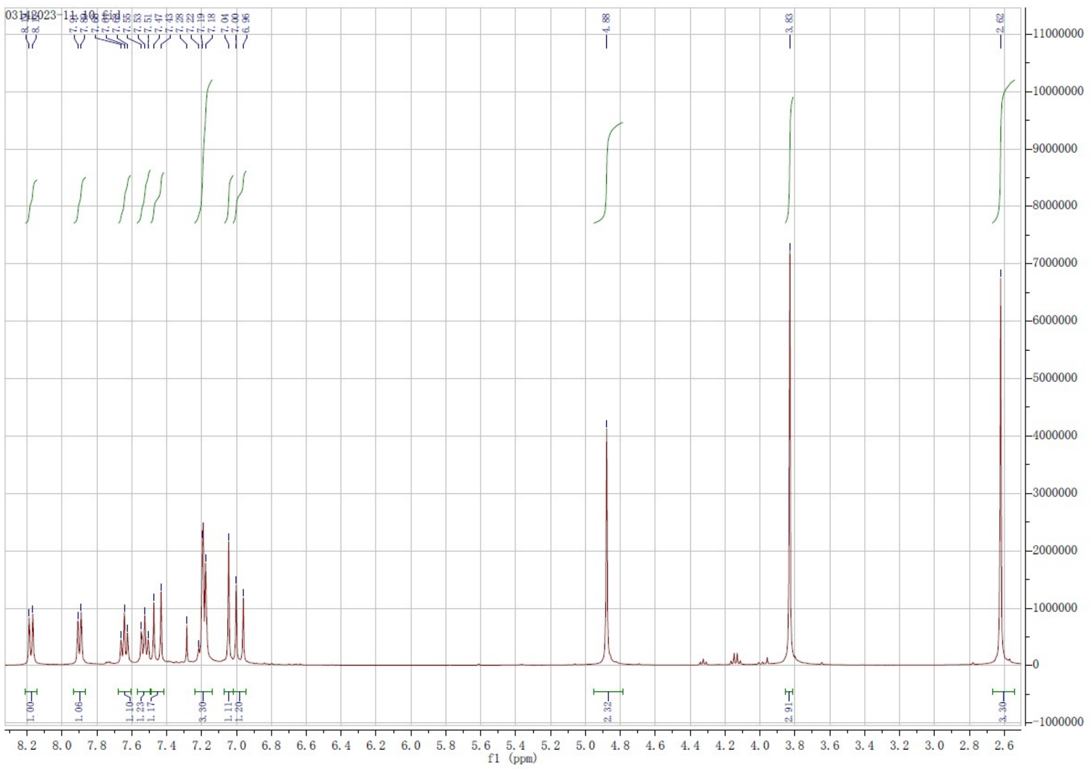

Figure S39. $^{1}$H NMR spectrum of compound **6t**

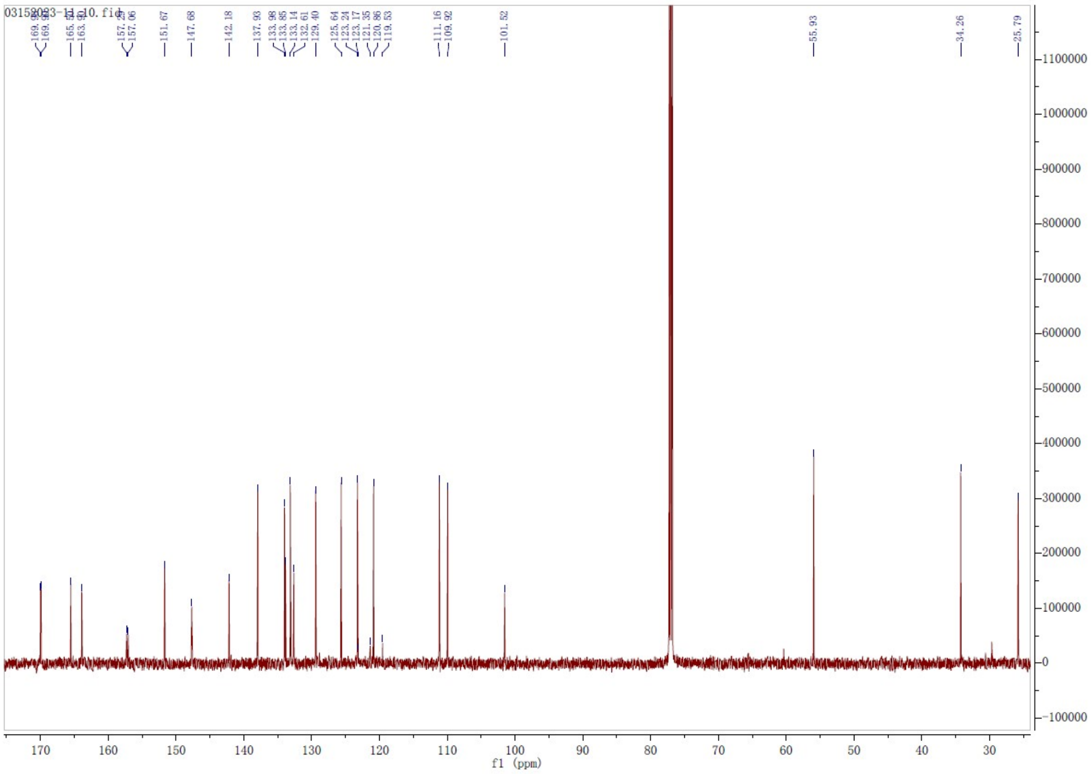

Figure S40. $^{13}$C NMR spectrum of compound **6t**

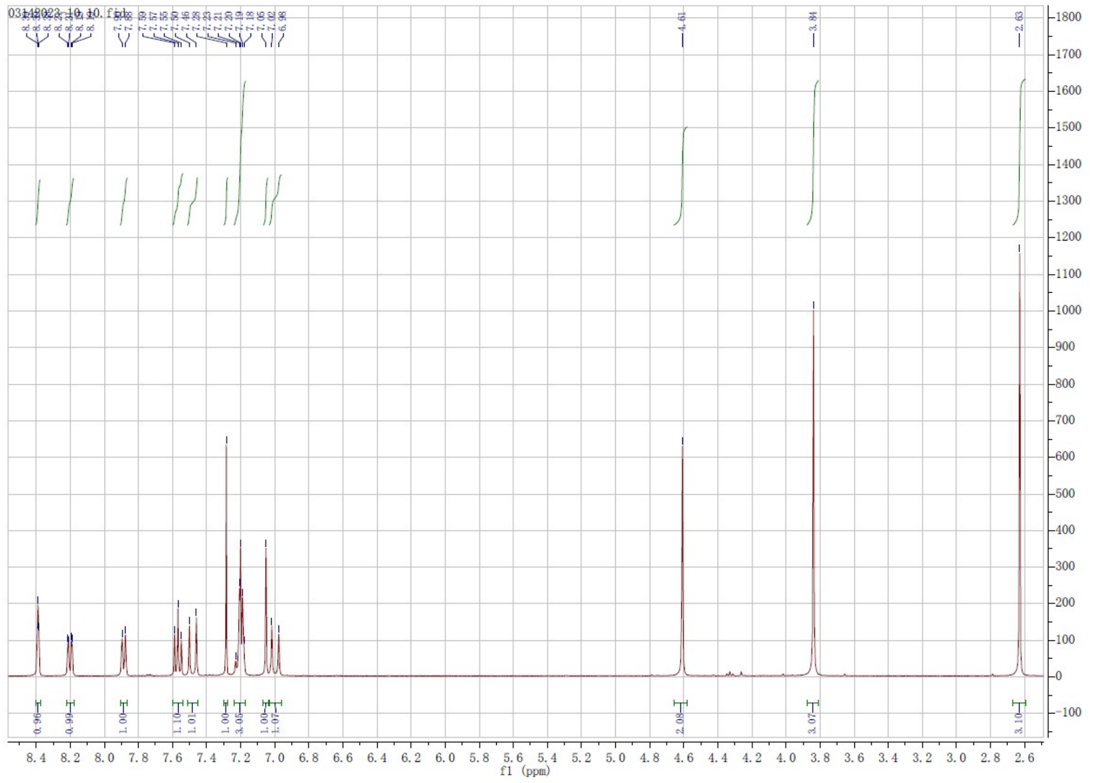

Figure S41. $^{1}H$ NMR spectrum of compound **6u**

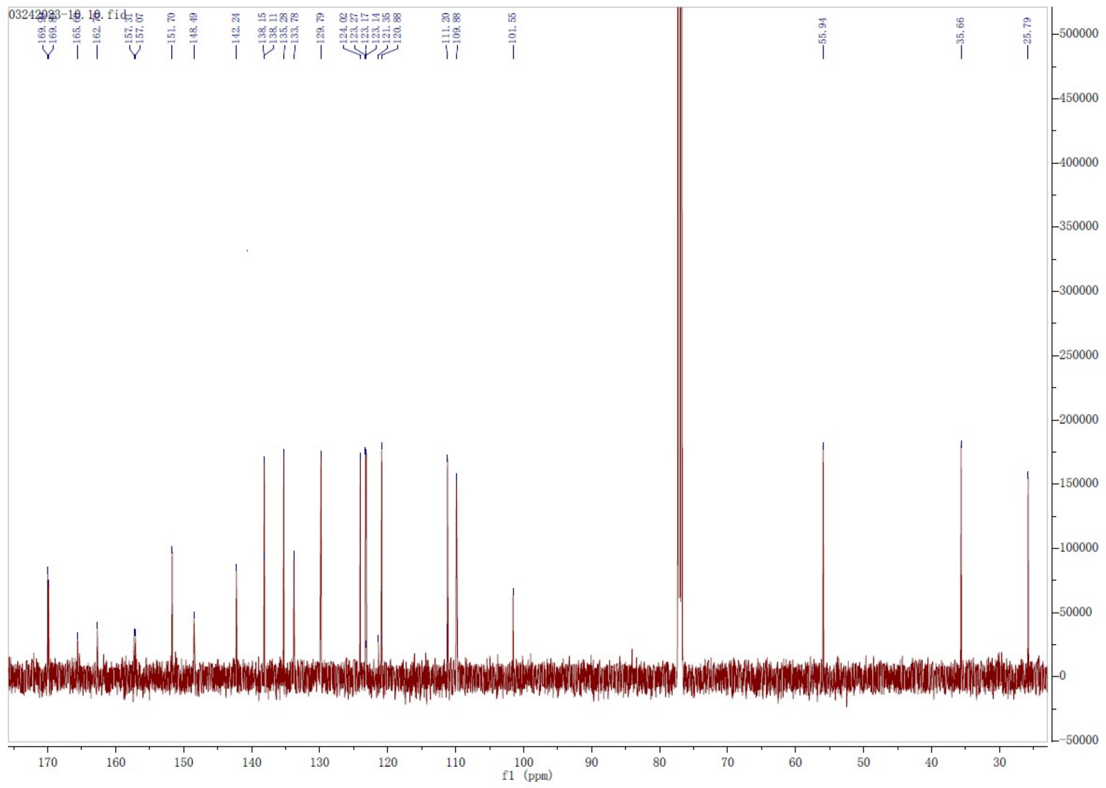

Figure S42. $^{13}C$ NMR spectrum of compound **6u**

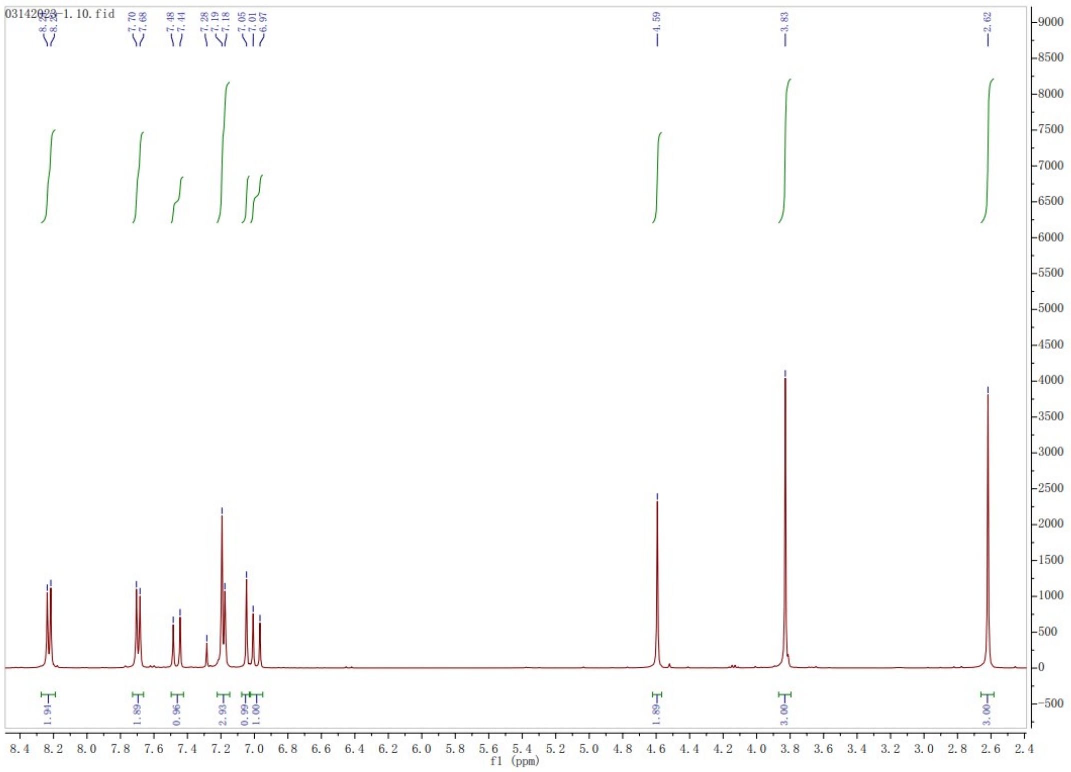

Figure S43. $^{1}$H NMR spectrum of compound **6v**

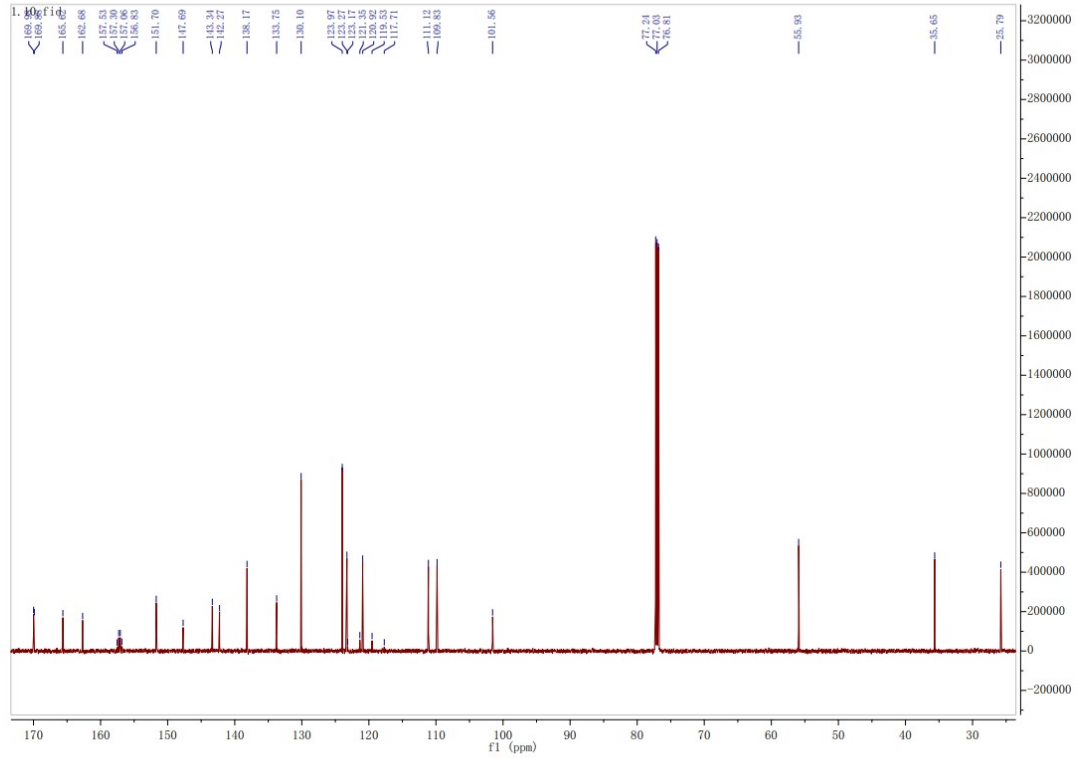

Figure S44. $^{13}$C NMR spectrum of compound **6v**

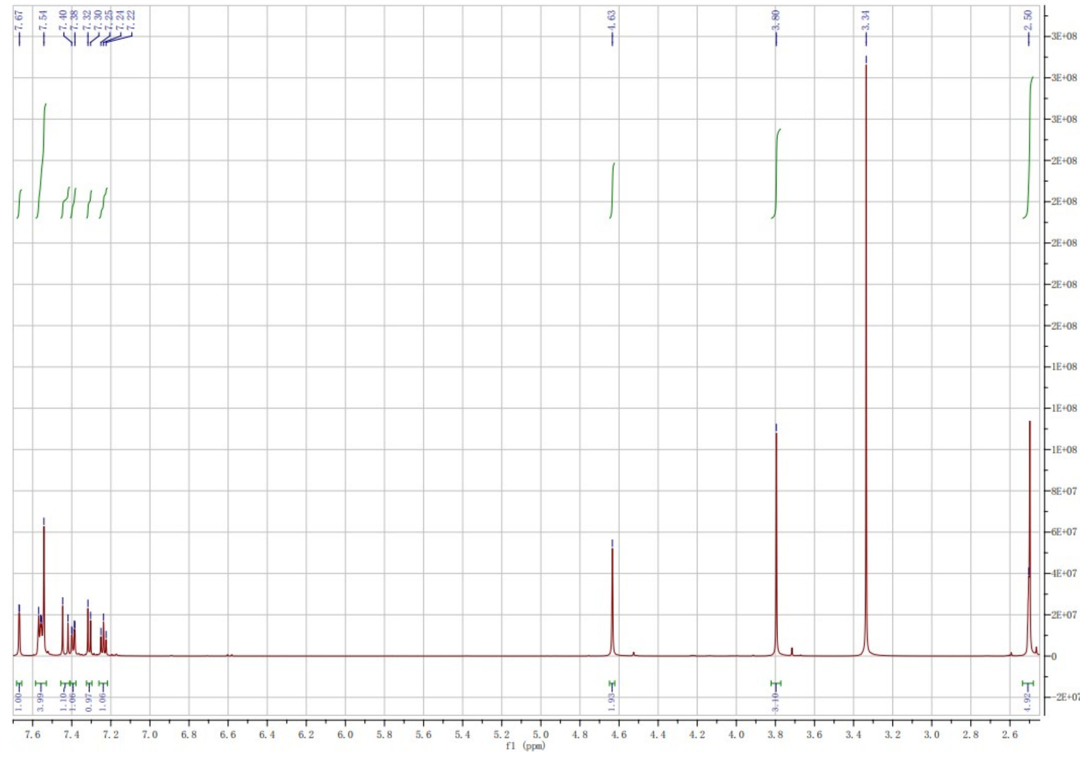


Figure S45. $^{1}H$ NMR spectrum of compound **6w**

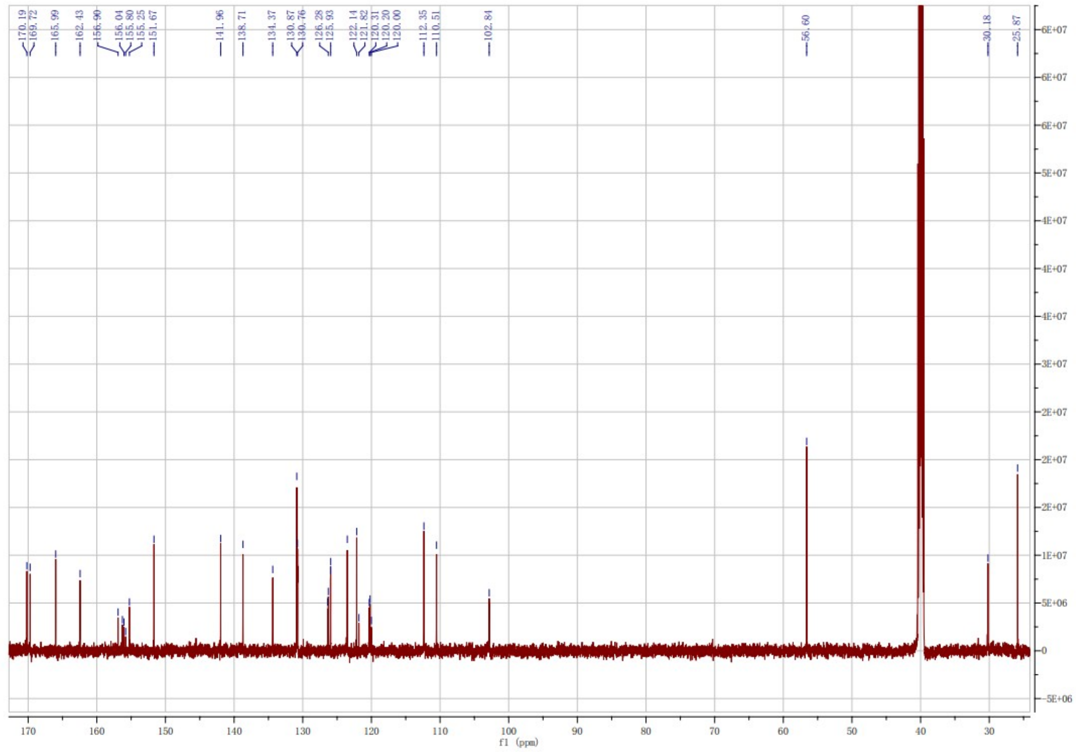


Figure S46. $^{13}C$ NMR spectrum of compound **6w**

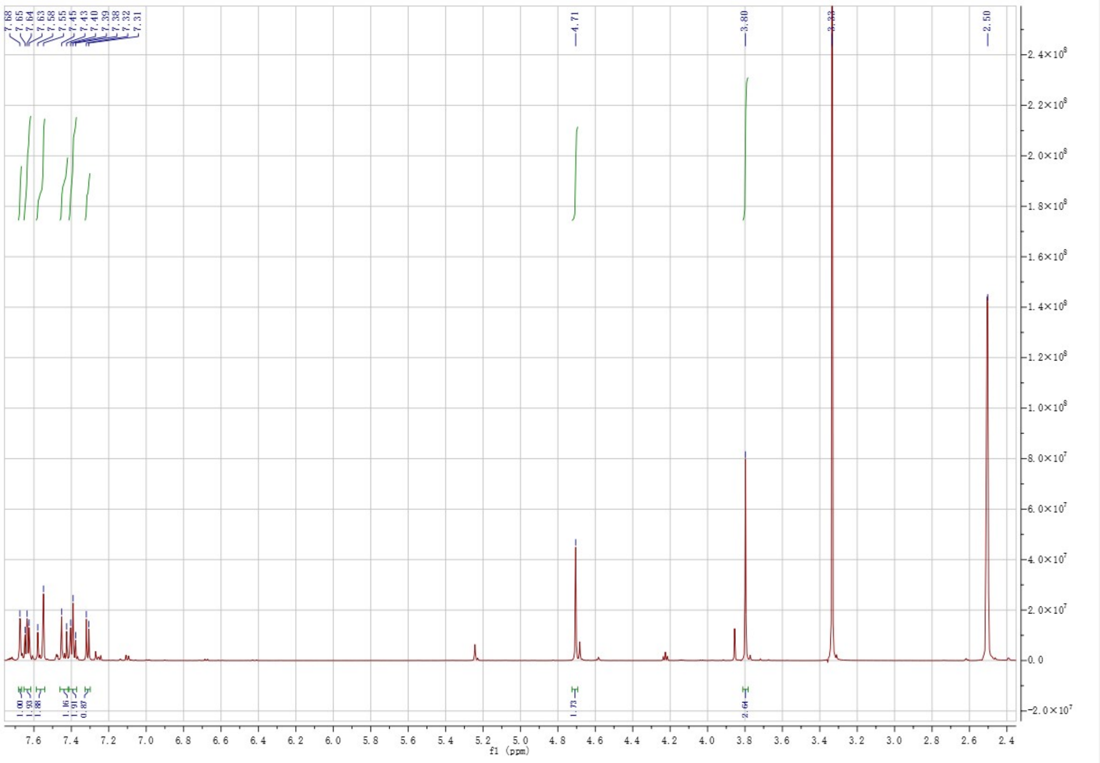

Figure S47. $^{1}$H NMR spectrum of compound **6x**

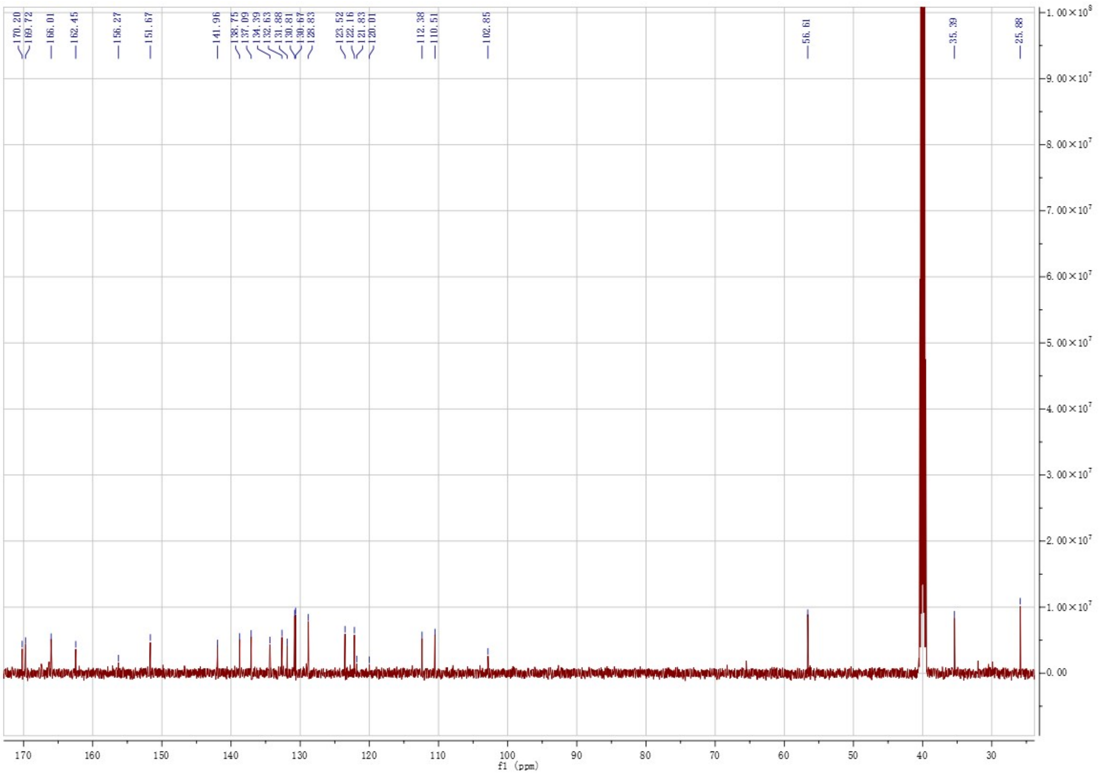

Figure S48. $^{13}$C NMR spectrum of compound **6x**
